# Rapidly improving ARDS differs clinically and biologically from persistent ARDS

**DOI:** 10.1186/s13054-024-04883-6

**Published:** 2024-04-22

**Authors:** Patricia L. Valda Toro, Andrew Willmore, Nelson E. Wu, Kevin L. Delucchi, Alejandra Jauregui, Pratik Sinha, Kathleen D. Liu, Carolyn M. Hendrickson, Aartik Sarma, Lucile P. A. Neyton, Aleksandra Leligdowicz, Charles R. Langelier, Hanjing Zhuo, Chayse Jones, Kirsten N. Kangelaris, Antonio D. Gomez, Michael A. Matthay, Carolyn S. Calfee

**Affiliations:** 1 Department of Medicine, Division of Pulmonary and Critical Care, University of Pennsylvania, Philadelphia, PA USA; 2 Division of Pulmonary, Critical Care, Allergy and Sleep Medicine, Department of Medicine, University of California, San Francisco, San Francisco, CA USA; 3 Department of Anesthesia, University of California, San Francisco, San Francisco, CA USA; 4 Department of Psychiatry, University of California, San Francisco, San Francisco, CA USA; 5 Division of Infectious Diseases, Department of Medicine, University of California, San Francisco, San Francisco, CA USA; 6 Chan Zuckerberg Biohub, San Francisco, CA USA; 7 Department of Anesthesiology, Washington University School of Medicine in St. Louis, St. Louis, MO USA; 8 Department of Critical Care Medicine, Toronto Western Hospital, Toronto, Canada; 9 Division of Hospital Medicine, Department of Medicine, University of California, San Francisco, San Francisco, CA USA; 10 Department of Internal Medicine, University of California San Francisco, San Francisco, USA

**Keywords:** Rapidly improving acute respiratory distress syndrome, Acute respiratory distress syndrome, Hypoinflammatory and hyperinflammatory ARDS phenotypes, Precision medicine, Prognostic and predictive enrichment of clinical trials

## Abstract

**Background:**

Rapidly improving acute respiratory distress syndrome (RIARDS) is an increasingly appreciated subgroup of ARDS in which hypoxemia improves within 24 h after initiation of mechanical ventilation. Detailed clinical and biological features of RIARDS have not been clearly defined, and it is unknown whether RIARDS is associated with the hypoinflammatory or hyperinflammatory phenotype of ARDS. The purpose of this study was to define the clinical and biological features of RIARDS and its association with inflammatory subphenotypes.

**Methods:**

We analyzed data from 215 patients who met Berlin criteria for ARDS (endotracheally intubated) and were enrolled in a prospective observational cohort conducted at two sites, one tertiary care center and one urban safety net hospital. RIARDS was defined according to previous studies as improvement of hypoxemia defined as (i) PaO_2_:FiO_2_ > 300 or (ii) SpO2: FiO_2_ > 315 on the day following diagnosis of ARDS (day 2) or (iii) unassisted breathing by day 2 and for the next 48 h (defined as absence of endotracheal intubation on day 2 through day 4). Plasma biomarkers were measured on samples collected on the day of study enrollment, and ARDS phenotypes were allocated as previously described.

**Results:**

RIARDS accounted for 21% of all ARDS participants. Patients with RIARDS had better clinical outcomes compared to those with persistent ARDS, with lower hospital mortality (13% vs. 57%; *p* value < 0.001) and more ICU-free days (median 24 vs. 0; *p* value < 0.001). Plasma levels of interleukin-6, interleukin-8, and plasminogen activator inhibitor-1 were significantly lower among patients with RIARDS. The hypoinflammatory phenotype of ARDS was more common among patients with RIARDS (78% vs. 51% in persistent ARDS; *p* value = 0.001).

**Conclusions:**

This study identifies a high prevalence of RIARDS in a multicenter observational cohort and confirms the more benign clinical course of these patients. We report the novel finding that RIARDS is characterized by lower concentrations of plasma biomarkers of inflammation compared to persistent ARDS, and that hypoinflammatory ARDS is more prevalent among patients with RIARDS. Identification and exclusion of RIARDS could potentially improve prognostic and predictive enrichment in clinical trials.

**Supplementary Information:**

The online version contains supplementary material available at 10.1186/s13054-024-04883-6.

## Background

Acute respiratory distress syndrome (ARDS) is a common cause of hypoxemic respiratory failure in critically ill patients and is a heterogeneous syndrome characterized by multiple clinical, physiological, and biological subphenotypes [1–4]. The failure of clinical trials exploring pharmacologic therapies for ARDS over the past fifty years has been attributed in part to such heterogeneity [1, 2, 5]. Rapidly improving ARDS (RIARDS) is a phenotype of ARDS in which hypoxemia (measured by PaO_2_ to FiO_2_ ratio) significantly improves within 24 h after initiation of mechanical ventilation [1, 6–10]. RIARDS has been described in between 7 and 15% of patients enrolled in randomized controlled trials who met ARDS criteria per Berlin definition and has been associated with better outcomes compared to persistent ARDS (defined as ARDS that persists over 24 h) [1, 3, 10].

The etiology of RIARDS is currently unknown, though several hypotheses have been proposed. Some have suggested that cases of RIARDS are likely “ARDS mimics,” such as autoimmune processes that are not characterized by the significant inflammation leading to alveolar damage [7]. Others have proposed that these rapidly improving cases actually represent atelectasis, since true inflammatory lung injury would not resolve that quickly [11, 12]. Relatively little is known about the detailed clinical and biological features of RIARDS. In this study, we sought to describe the clinical and biological profile of RIARDS in order to better understand its pathophysiology and potential relevance to future clinical trials and clinical practice. We hypothesized that RIARDS would be characterized by a less severe clinical course compared to persistent ARDS, and that biomarkers of inflammation and lung endothelial and epithelial injury would differ between RIARDS and persistent ARDS. We also hypothesized that RIARDS would be associated with the hypoinflammatory ARDS phenotype that has been previously described by our group and others [3, 13–16]. Characterization and early identification of RIARDS could potentially improve prognostic and predictive enrichment of ARDS clinical trials, which have traditionally been impacted by the heterogeneity of this disease.

## Methods

We analyzed data from 215 ARDS patients enrolled in the Early Assessment of Renal and Lung Injury (EARLI) cohort from November 2008 to May 2018. EARLI is a dual-center prospective observational cohort of critically ill patients at the University of California San Francisco Medical Center and Zuckerberg San Francisco General Hospital; details of inclusion and exclusion criteria as well as full details of informed consent processes have been previously published [16, 17]. For this study, we analyzed data from participants who met Berlin criteria for ARDS on day 1 or 2 of the study, were endotracheally intubated at the time of meeting Berlin criteria, and had plasma biomarker measurements available [18]. ARDS adjudication was determined by consensus of two board-certified physicians who reviewed all clinical data and chest radiographs for enrolled patients, blinded to biomarker data. ARDS criteria were met per Berlin definition based on oxygenation (PaO_2_: FiO_2_ < 300 or SpO_2_: FiO_2_ < 315), timing of symptoms, and radiographic evidence of bilateral opacities. The lowest PaO_2_: FiO_2_ ratio recorded in the day was used in the analysis of this study. ARDS risk factors and non-cardiogenic pulmonary edema versus cardiogenic pulmonary edema (or mixed) were determined by physician review of clinical data (Table 1). The day of ARDS diagnosis was designated as day 1 for all subsequent analysis of this study.

Rapidly improving ARDS was defined according to previous studies as either (i) PaO_2_: FiO_2_ > 300 or (ii) Spo2: FiO_2_ > 315 on the day following diagnosis of ARDS (day 2) or (iii) unassisted breathing by day 2 and for the next 48 h (defined as absence of endotracheal intubation on day 2 through day 4) [1]. We intended to select patients with overall improvement in hypoxemia; therefore, four patients who initially met criteria for RIARDS but died on the day of extubation or within twenty-four hours thereafter were instead classified as persistent ARDS. Three out of the four patients re-classified as persistent ARDS had a PaO_2_: FiO_2_ < 300 on day 2 and were palliatively extubated that day after transitioning to comfort care measures only. One of the four patients initially met criteria for RIARDS by having a PaO_2_: FiO_2_ > 300 on day 2 but died on day 3 from a pulseless electric activity arrest secondary to hypoxemia, and thus was re-classified as persistent ARDS (Additional file 1: Fig. E1).

Clinical characteristics were analyzed from comprehensive data that were collated into a database by direct extraction from electronic medical records and manually by trained study coordinators and physician investigators. Medical history was obtained via manual review of clinical documentation as described in Additional file 1, 2, and 3. In-hospital mortality, ICU free days and ventilator free days were calculated within 28 days of ARDS diagnosis. Allocation to hyperinflammatory versus hypoinflammatory subphenotype was previously done using latent class analysis [16]. In a sensitivity analysis to address the question if the degree of hypoxia at time of ARDS diagnosis affects the likelihood of rapid ARDS resolution, we focused on severe ARDS (defined by a PaO_2_: FiO_2_ equal to or less than 100 at time of enrollment) and compared the clinical and molecular characteristics between patients with RIARDS versus persistent disease. A sensitivity analysis comparing RIARDS and persistent disease among cases allocated to the hyperinflammatory phenotype was also performed.

### Biomarkers

We analyzed plasma markers of inflammation, lung epithelial injury, endothelial injury and coagulation that have been previously associated with the pathophysiology of ARDS [3, 16, 19–32]. These plasma biomarkers were measured on samples collected within 1 day of meeting ARDS criteria. Details on specific biomarkers measured and assay procedures are included in Additional file 1: Table E13.

### Statistical analysis

Basic two-group comparisons between the two cohorts were done using the Mann–Whitney U test, Pearson’s *χ*^2^ test or Fisher’s exact test, as appropriate. Data were analyzed using Python 3 (Jupyter 6.4.5) and STATA SE 18. Statistical significance was defined by a *p* value less than or equal to 0.05. Bonferroni correction was used when analyzing statistical significance for multiple biomarkers in order to avoid risk of type I error, and the resulting corrected p value is denoted as an adjusted *p* value in Table 3.

## Results

Two hundred and fifteen patients with Berlin ARDS were included in the analysis. Of those, forty-six (21%) met criteria for RIARDS. Thirteen patients met criteria based on a PaO_2_: FiO_2_ ratio greater than 300 on day 2, two patients met criteria based on a SpO2: FiO_2_ ratio greater than 315 on day 2, and thirty-one met criteria due to achieving unassisted breathing by day 2 (see Additional file 1: Figure E1).

### Clinical characteristics of RIARDS versus persistent ARDS

Baseline clinical characteristics for patients stratified by the presence or absence of RIARDS are summarized in Table 1. Non-Hispanic ethnicity was more common among patients with RIARDS. Otherwise, age, gender and race did not differ significantly between RIARDS and persistent ARDS, nor did primary ARDS risk factor. In terms of comorbidities, cirrhosis was more commonly identified in persistent ARDS (12% vs. 2%, *p* = 0.05, Additional file 1: Tables E1 and E14). Hypertensive crisis at time of enrollment was more common in patients with RIARDS compared to those with persistent ARDS (Table 2).

**Table 1:** Baseline clinical characteristics for patients stratified by presence or absence of RIARDS. Mild ARDS was more commonly observed in RIARDS. The prevalence of vasopressor-dependent shock on day 1 was lower among patients with RIARDS. Patients with RIARDS had a lower 28-day mortality and more ICU-free days compared with persistent ARDS

	Persistent ARDS N = 169	RIARDS N = 46	Total N = 215	*p* value
*General patient characteristics*				
Age, median (IQR)	66 (54, 79)	63 (47, 78)	66 (53, 79)	0.41
BMI, median (IQR)	25 (22, 31)	24 (22, 27)	25 (22, 30)	0.22
Gender				
Male, n (%)	101 (60)	29 (63)	130 (60)	0.92
Female, n (%)	66 (39)	17 (37)	83 (39)	
Transgender, n (%)	2 (1)	0 (0)	2 (1)	
Race				
Caucasian, n (%)	76 (45)	30 (65)	106 (49)	0.12
African American, n (%)	26 (15)	5 (11)	31 (14)	
Asian, n (%)	44 (26)	8 (17)	52 (24)	
Pacific Islander, n (%)	1 (1)	0 (0)	1 (0.5)	
Native American, n (%)	0 (0)	0 (0)	0 (0)	
Other, n (%)	21 (12)	2 (4)	23 (11)	
Unknown, n (%)	1 (1)	1 (2)	2 (1)	
Ethnicity				
Hispanic, n (%)	25 (15)	1 (2)	26 (12)	**0.02**
Non-Hispanic, n (%)	143 (85)	44 (96)	187 (87)	
Missing, n (%)	1 (1)	1 (2)	2 (1)	
Hospital setting				
Tertiary care center, n (%)	113 (67)	36 (78)	149 (69)	0.14*
Urban safety net hospital, n (%)	56 (33)	10 (22)	66 (31)	
Primary ARDS risk factor				
Pneumonia, n (%)	59 (35)	19 (41)	78 (36)	0.89
Sepsis, n (%)	66 (39)	14 (30)	80 (37)	
Aspiration, n (%)	31 (18)	10 (22)	41 (19)	
Transfusions, n (%)	5 (3)	1 (2)	6 (3)	
Drug overdose, n (%)	1 (1)	0 (0)	1 (0.5)	
Other, n (%)	2 (1)	1 (2)	3 (1)	
None, n (%)	4 (2)	1 (2)	5 (2)	
Missing, n (%)	1(0.6)	0 (0)	1 (0.5)	
Severity of ARDS at enrollment by PaO_2_/ FiO_2_				
Mild, n (%)	23 (14)	21 (46)	44 (20)	< 0.001*
Moderate, n (%)	67 (40)	18 (39)	85 (40)	
Severe, n (%)	79 (47)	7 (15)	86 (40)	
“Pure” ARDS (non-cardiogenic edema) versus mixed cardiogenic and non-cardiogenic edema†				
“Pure” ARDS, n (%)	123 (73)	31 (67)	154 (72)	0.47*
Mixed etiology of pulmonary edema, n (%)	46 (27)	15 (33)	61 (28)	
CXR quadrants involved, qualifying CXR				
Two, n (%)	44 (26)	16 (35)	60 (28)	0.21
Three, n (%)	54 (32)	18 (39)	72 (33)	
Four, n (%)	67 (40)	11 (24)	78 (36)	
Missing, n (%)	4 (2)	1 (2)	5 (2)	
Severity of disease within 1 day of ARDS diagnosis				
APACHE III score, median (IQR)	135 (109, 160)	94 (81, 133)	126 (99, 154)	** < 0.001**
Vasopressor-dependent shock, n (%)	136 (80)	28 (61)	164 (76)	0.01*
RRT-requiring renal failure, n (%)	27 (16)	4 (9)	31 (14)	0.41
Clinical outcomes				
Hospital mortality at 28 days, n (%)	96 (57)	6 (13)	102 (47)	** < 0.001**
ICU-free days, median (IQR)	0 (0, 18)	24 (21, 25)	2 (0, 22)	** < 0.001**
Ventilator-free days, median (IQR)	0 (0, 21)	26 (25, 26)	0 (0, 24)	** < 0.001**

*p* values that met criteria for statistical signifcance (*p* ≤ 0.05) were bolded

Mann–Whitney U Test or Fisher’s test was used to determine statistical significance unless otherwise specified

^*^Pearson’s chi-square test was used to determine statistical significance

^†^Determined by two board-certified physicians who independently reviewed radiological and clinical data

**Table 2:** Clinical parameters were compared between RIARDS and persistent ARDS. Ventilatory parameters significantly differed between persistent ARDS and RIARDS. Steroids were more commonly administered to persistent ARDS patients compared to RIARDS over the first 48 hours of ARDS diagnosis. Bacteremia was more common in persistent ARDS

		Persistent ARDS N = 169	RIARDS N = 46	*p* value
*Clinical parameters*				
Ventilatory parameters on day 1 of ARDS diagnosis				
Plateau pressure (cmH_2_O), median (IQR)†	24 (20, 28)		20 (17, 21)	**0.0003**
PIP (cmH_2_O), median (IQR)†	31 (26, 37)		26 (22, 31)	**0.001**
FiO_2_ (%), median (IQR) †	1 (0.6, 1)		0.6 (0.5, 0.9)	**0.001**
PEEP (cmH_2_O), median (IQR)	5 (5, 10)		5 (5, 8)	**0.01**
paCO_2_ (mmHg), median (IQR)	41 (33, 52)		44 (33, 49)	0.84
Arterial pressure on day 1 of ARDS diagnosis				
MAP (mmHg), median (IQR)	78 (68, 93)		76 (67, 90)	0.50
SBP (mmHg), median (IQR)	113 (98, 132)		110 (99, 126)	0.52
Medications received within 48 h of ARDS diagnosis				
Steroids, n (%)	65 (38)		6 (13)	0.001*
Diuretics, n (%)	35 (21)		13 (28)	0.28*
Antibiotics, n (%)	161 (95)		45 (98)	0.44*
Antivirals, n (%)	41 (24)		9 (20)	0.50*
Antifungals, n (%)	17 (10)		1 (2)	0.13
Microbiology within 48 h of ARDS diagnosis				
Pulmonary infection				
Bacterial, n (%)	33 (20)		6 (13)	0.31*
Viral, n (%)	14 (8)		7 (15)	0.16*
Fungal, n (%)	0 (0)		0 (0)	–
Bacteremia, n (%)	30 (18)		2 (4)	**0.02**

Mann–Whitney U Test or Fisher’s test was used to determine statistical significance unless otherwise specified

*p* values that met criteria for statistical signifcance (*p* ≤ 0.05) were bolded

*PIP* peak inspiratory pressure; *FiO*_*2*_ fraction of inspired oxygen; *PEEP* positive end-expiratory pressure; *paCO*_*2*_ partial pressure of carbon dioxide in arterial blood gas; *MAP* mean arterial pressure; *SBP* systolic blood pressure

^*^Pearson’s chi-square test was used to determine statistical significance

^†^Missing values for some patients as indicated in supplement table eight

Mild ARDS (defined by a PaO_2_: FiO_2_ of 201–300 mm Hg at enrollment) was more commonly observed in RIARDS (21/46 patients; [46%]) compared to persistent ARDS (23/169 patients; [14%]; *p* < 0.001). Conversely, a higher prevalence of severe ARDS (defined by a PaO_2_: FiO_2_ less than or equal to 100) was appreciated in persistent ARDS (79/169 [47%] vs. 7/46 [15%] in RIARDS; Table 1). The number of quadrants involved on chest radiograph did not differ in RIARDS versus persistent ARDS, nor did the concomitant presence of physician-adjudicated mixed cardiogenic and non-cardiogenic pulmonary edema (Table 1).

APACHE III scores were on average lower among patients with RIARDS (median 94 vs. 135 in persistent ARDS; *p* < 0.001), and prevalence of vasopressor-dependent shock on day 1 was lower as well (61% vs. 80%, *p* = 0.01). The less severe nature of RIARDS was further appreciated by the lower 28-day mortality (13% vs. 57%, *p* < 0.001) and more ICU-free days, compared with persistent ARDS (median 24 vs. 0 days, *p* < 0.001) (Table 1).

Ventilatory parameters on day 1 of ARDS diagnosis were compared between RIARDS and persistent ARDS (Table 2 and Additional file 1: Table E12). Positive end-expiratory pressure (PEEP), peak inspiratory pressure (PIP), plateau pressure and FiO_2_ were each significantly higher among persistent ARDS cases compared to RIARDS (Table 2), suggesting greater severity of lung injury in persistent patients. Steroids were more commonly administered to persistent ARDS patients compared to RIARDS over the first 48 h of ARDS diagnosis (*p* = 0.001, Table 2). The type of steroid administered per group is described in Table 3. There were no significant differences in diuretics or antimicrobials use by RIARDS status (Table 2). Bacteremia was more common in persistent ARDS (18% vs. 4%; *p* = 0.02). Microbiologically confirmed pulmonary infection, including bacterial and viral, was equally prevalent in each group (Table 2).

**Table 3:** Differences in plasma biomarker levels were found when comparing patients with RIARDS to patients with persistent ARDS. Analyses adjusting for multiple comparisons showed that plasma IL-6, IL-8 and PAI-1 were significantly lower in patients with RIARDS compared to those with persistent ARDS

Biomarker	Persistent ARDS N = 169 Median (IQR)	RIARDS N = 46 Median (IQR)	*p* value	Bonferroni adjusted *p* value^‡^
*Biomarkers in RIARDS versus persistent ARDS*				
Inflammation mediators				
IL-8 (pg/ml)	62 (17, 453)	16 (7, 68)	**0.0001**	**0.001**
IL-6 (pg/ml)	462 (83, 4704)	80 (33, 474)	**0.0002**	**0.003**
TNF alpha (pg/ml)	88 (66, 147)	71 (62, 90)	**0.006**	0.08
IL-10 (pg/ml)	38 (26, 103)	28 (23, 44)	**0.007**	0.09
IFN gamma (pg/ml)	113 (93, 145)	106 (82, 128)	0.05	0.62
IL-1Beta (pg/ml)	78 (64, 105)	73 (56, 91)	0.05	0.71
CX3CL1 (pg/ml)	13,180 (11,488, 15,440)	11,974 (10,688, 14,626)	0.09	1.0
sTNFR-1 (pg/ml)	5273 (2516, 10,436)	4563 (1482, 10,125)	0.20	1.0
*Lung injury*				
Alveolar type I				
sRAGE (pg/ml)	4579 (2495, 7535)	3657 (2081, 7921)	0.24	1.0
*Endothelial cell*				
Ang-2 (pg/ml)	9059 (4531, 16,221)	5569 (3397, 9435)	**0.005**	0.06
ICAM-1 (pg/ml)	677,335 (332,593, 1,544,076)	456,391 (280,394, 714,185)	**0.01**	0.14
Coagulation				
PAI-1 (ng/ml)	18 (5, 50)	5 (3, 11)	**0.0001**	**0.001**
Protein C (% control)	69 (33, 129)	87 (58, 158)	**0.04**	0.51

*p* values that met criteria for statistical signifcance (*p* ≤ 0.05) were bolded

IL-8: Interleukin-8; IL-6: Interleukin-6; TNF alpha: tumor necrosis factor alpha; IL-10: Interleukin-10; IFN gamma: interferon gamma; IL-1Beta: interleukin-1 Beta; CX3CL1: gene encoding chemokine ligand 1; sTNFR-1: soluble tumor necrosis factor receptor-1; sRAGE: soluble receptor for advanced glycation end-products; Ang-2: angiopoietin-2; ICAM-1: intercellular adhesion molecule 1; PAI-1: plasminogen activator inhibitor 1

^‡^Mann–Whitney U Test was used to calculate uncorrected alpha. Adjusted p value was obtained using Bonferroni correction

### Biological profile of RIARDS versus persistent ARDS

**Fig. 1:**
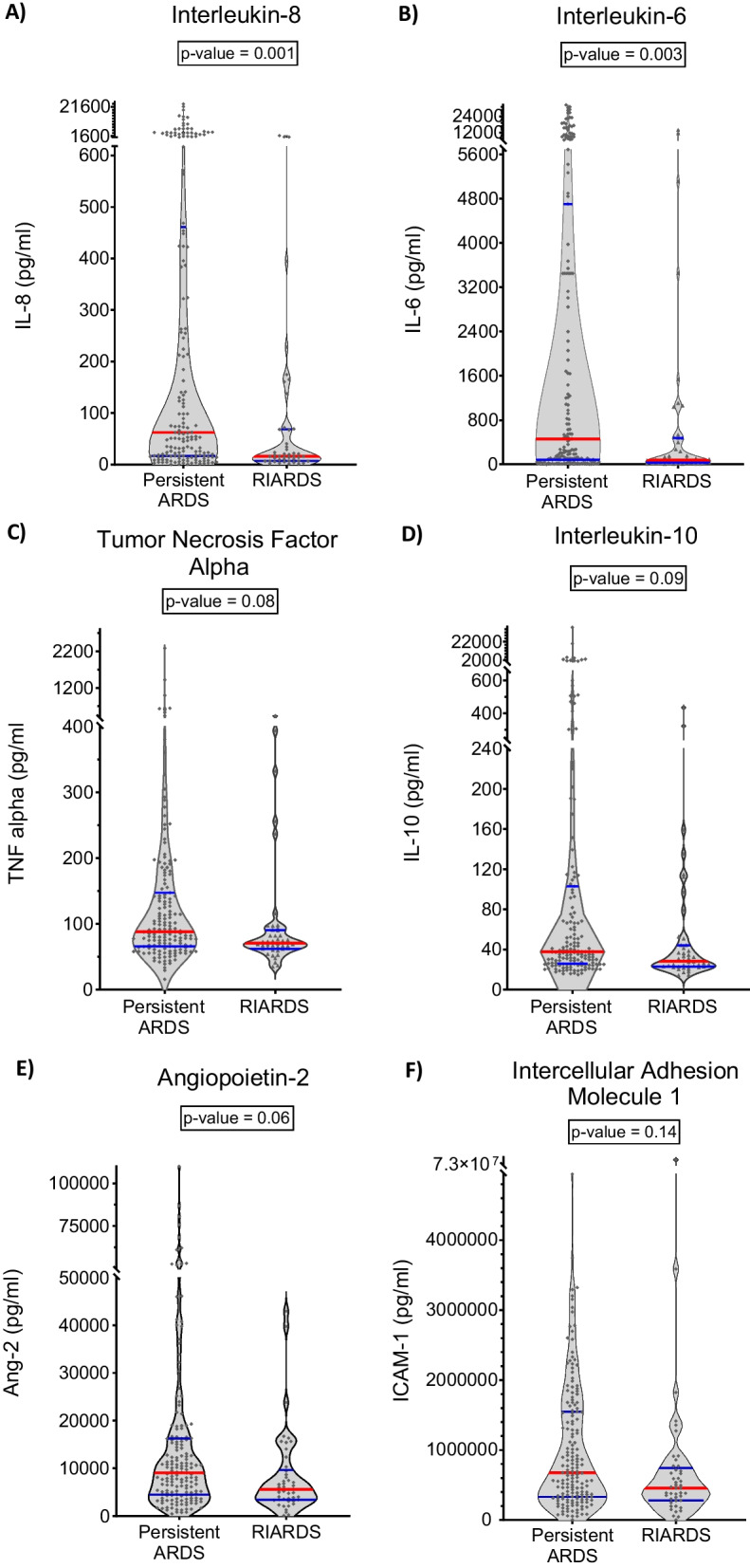

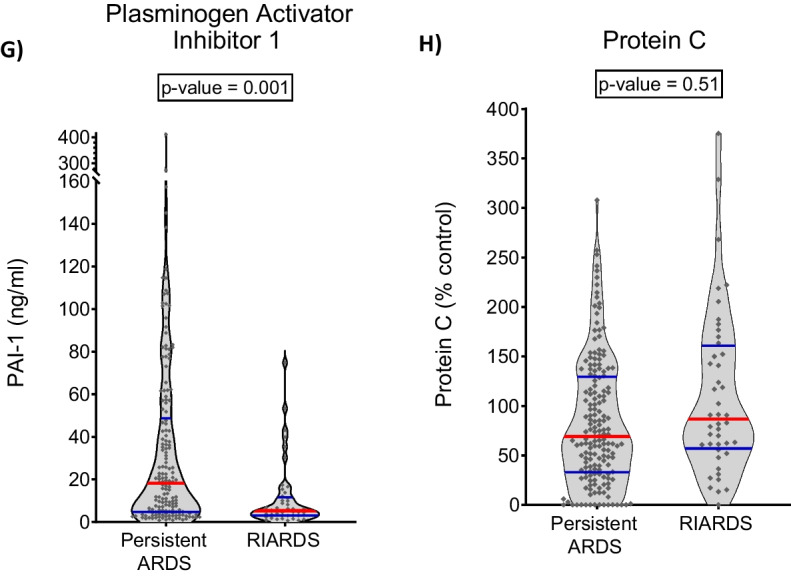
Biomarker Distribution in RIARDS versus Persistent ARDS. Plasma concentrations of interleukin-8 (**A**), interleukin-6 (**B**), plasma concentrations of tumor necrosis factor alpha (**C**), interleukin-10 (**D**), angiopoietin-2 (**E**), and intercellular adhesion molecule 1 (**F**) were not significantly different between patients with RIARDS and persistent ARDS and plasminogen activator inhibitor 1 (**G**) were significantly different between patients with RIARDS and persistent ARDS. Red line represents median value. Blue lines represent IQR

Differences in plasma biomarker levels were found when comparing patients with RIARDS to patients with persistent ARDS (Table 3). In unadjusted analysis, plasma concentration of interleukin (IL) 8, IL-6, IL-10, tumor necrosis factor alpha (TNF alpha), angiopoietin-2 (Ang-2), intercellular adhesion molecule 1 (ICAM-1) and plasminogen activator inhibitor 1 (PAI-1) were significantly lower in patients with RIARDS compared to those with persistent ARDS. Concentrations of Protein C were significantly higher in RIARDS. In analyses adjusting for multiple comparisons, plasma IL-6, IL-8 and PAI-1 were significantly lower in patients with RIARDS compared to those with persistent ARDS (Table 3 and Fig. 1). Notably, plasma levels of Soluble Receptor for Advanced Glycation End Products (sRAGE), commonly used as a biomarker of alveolar epithelial injury, were similar in RIARDS and persistent ARDS (Table 3).

### Association between RIARDS and latent ARDS phenotypes

**Fig. 2:**
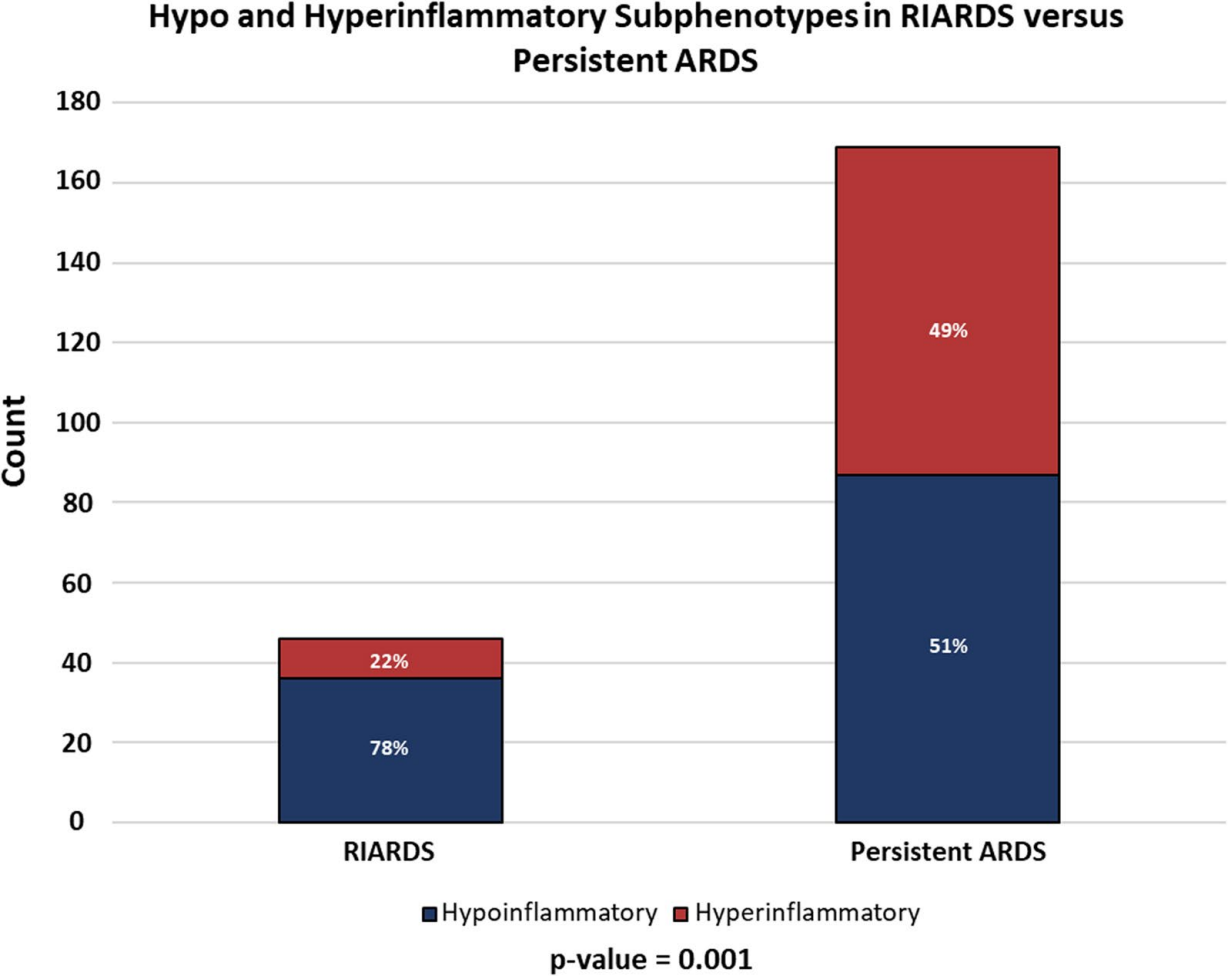
The hypoinflammatory subphenotype of ARDS was more common than the hyperinflammatory subphenotype in patients with RIARDS compared to persistent ARDS

Overall, 92 of the 215 ARDS patients (43%) were classified into the hyperinflammatory phenotype, with the remaining 123 (57%) in the hypoinflammatory phenotype. The hypoinflammatory phenotype of ARDS was significantly more common than the hyperinflammatory phenotype in patients with RIARDS (78% hypo- vs. 22% hyper- in RIARD, compared to 51% and 49%, respectively, in persistent ARDS; *p* = 0.001) (Fig. 2).

### Sensitivity analysis: severe RIARDS

We identified seven patients who had severe ARDS (i.e., PaO_2_: FiO_2_ less than or equal to 100) at time of enrollment and meeting criteria for RIARDS, as compared to 79 patients with severe persistent ARDS. All seven of the severe RIARDS patients were extubated the day after enrollment. One of these seven patients died six days after extubation, while the rest were discharged alive. The majority of these patients (4/7; 57%) had aspiration as their ARDS risk factor. Differences in prevalence of vasopressor-dependent shock and in clinical outcomes between severe RIARDS versus severe persistent ARDS mirrored the pattern observed in the overall cohort (Additional file 1: Table E4). Comorbidities, concomitant medical conditions and ventilatory parameters did not differ significantly between RIARDS and persistent disease among those with severe ARDS (Additional file 1: Tables E5, E6, and E7). Plasma inflammatory biomarkers were significantly higher in those with severe persistent ARDS compared to severe RIARDS, as shown in Additional file 1: Table E8.

### Sensitivity analysis: hyperinflammatory ARDS

We identified ten patients who were both classified into the hyperinflammatory phenotype and met criteria for RIARDS. Similar to the results seen in the overall cohort, compared to patients with hyperinflammatory persistent ARDS, patients with hyperinflammatory RIARDS had significantly lower in-hospital mortality at 28 days (20% vs. 68%, *p* = 0.01) and higher ICU-free days (median 24 vs. 0, *p* < 0.001). ARDS severity at time of enrollment differed significantly among those with the hyperinflammatory phenotype, with severe hypoxemia commonly seen in persistent ARDS and not appreciated in RIARDS (60% vs. 0%, *p* < 0.001) (Additional file 1: Table E9).

APACHE III scores were on average lower among patients with hyperinflammatory RIARDS compared to hyperinflammatory persistent ARDS (median 110 vs. 153, *p* = 0.02). However, contrary to results seen in the overall cohort, vasopressor-dependent shock on day 1 was equally prevalent (100% in RIARDS versus 99% in persistent ARDS, *p* = 1.0) (Additional file 1: Table E9). Renal failure requiring renal replacement therapy was more prevalent among those with hyperinflammatory persistent ARDS compared to hyperinflammatory RIARDS (30% vs. 0%, *p* = 0.03), which differed from the equal prevalence seen between both groups in the overall cohort (Additional file 1: Table E9). Comorbidities, microbiology and medications received did not differ between each group (Additional file 1: Table E10).

Ventilatory parameters were similar between each group, with the exception of delivered FiO_2_ which was significantly higher among persistent ARDS cases (median 1 vs. 0.6, *p* = 0.03) (Additional file 1: Table E10). No significant differences were found in plasma inflammatory biomarker concentration between patients with RIARDS and persistent ARDS in this sensitivity analysis (Additional file 1: Table E11).

## Discussion

Rapidly improving ARDS has been appreciated since at least the year 2000, but there has been little research to date characterizing the biological profile of these patients [1]. This study reports the novel finding that RIARDS is characterized by lower concentrations of plasma biomarkers of inflammation compared to persistent ARDS. Also consistent with this data, we found that the hypoinflammatory ARDS phenotype is more prevalent among patients with RIARDS than those with persistent disease. Furthermore, our study identified a high prevalence of RIARDS in a multicenter, “real world” observational cohort (as opposed to clinical trials) and confirms prior reports regarding the more benign clinical course of these patients [1, 10].

RIARDS accounted for 21% of all ARDS patients studied in this cohort. This prevalence is higher than the previously reported 16% appreciated in the observational LUNG SAFE study, and in randomized controlled trials (which has ranged from 7.3 to 15.2%) [1, 33]. The higher prevalence in our cohort might be explained by differences in our inclusion criteria or our approach to phenotyping. This cohort includes patients diagnosed with ARDS per Berlin definition without any other exclusion criteria other than undergoing mechanical ventilation via an endotracheal tube (as opposed to more selective criteria which are often used in clinical trials). On the other hand, past therapeutic clinical trials in which RIARDS was previously described, excluded patients with conditions such as severe chronic respiratory disease, morbid obesity, lung transplant, severe chronic liver disease, or chronic dialysis, all of which were included in our cohort, potentially increasing disease severity in our studied population. In addition, we have taken a detailed approach to clinical phenotyping including two physician review of all chest radiographs and clinical data on enrolled patients for ARDS ascertainment. These findings indicate that RIARDS is quite common in a “real-world” ICU population and highlight the heterogeneity that is captured by the Berlin definition of ARDS.

Consistent with previously reported data, we found that patients with RIARDS have a more favorable clinical course with lower mortality and higher ICU-free days [1]. These improved outcomes were further appreciated in our sensitivity analyses focusing on severe ARDS and the hyperinflammatory ARDS phenotype, in which we found that patients with RIARDS still have shorter ICU admissions and lower mortality. Given the prevalence and clinical outcomes of RIARDS, inclusion of these patients in clinical trials may make it difficult to identify therapeutic benefits, since these patients appear to improve so quickly.

The etiology of RIARDS has been debated in the past. Some have argued that RIARDS is likely an “ARDS mimic” [7]—in other words, that the fast resolution of hypoxemia in these cases was not reflective of the diffuse alveolar damage frequently observed in ARDS but was instead generated by other transient causes such as atelectasis or cardiogenic pulmonary edema [4, 7, 34]. In this study, we found that patients with RIARDS more commonly had hypertensive emergency at the time of enrollment, suggesting the possibility that hypoxemia in these cases was at least in part related to cardiogenic pulmonary edema. However, additional clinical data did not provide strong evidence to favor cardiogenic pulmonary edema as a leading etiology of RIARDS. Review of radiographic data by board-certified physicians showed that “pure” ARDS versus ARDS with concomitant heart failure and/or volume overload were equally prevalent between patients with RIARDS and persistent disease. Furthermore, volume overload was considered to be equally prevalent in patients with RIARDS compared to persistent ARDS by the treating physician based on our manual review of clinical documentation. Finally, comorbidities that predispose to volume overload and cardiogenic edema, such as renal disease, ventricular dysfunction, and hypertension, were equally common between patients with RIARDS and persistent ARDS.

Although the significant differences in ventilatory support needed between RIARDS and persistent ARDS appear to reflect physiologic differences in lung injury and alveolar compliance, no significant differences were appreciated between plasma levels of soluble receptor for advanced glycation end-products (sRAGE), which has served as an indicator of alveolar epithelial injury. sRAGE has been helpful in distinguishing pulmonary edema secondary to acute lung injury from edema caused by elevated capillary hydrostatic (i.e., cardiogenic) pressure [23, 35, 36]. Therefore, the similarity in sRAGE levels between RIARDS and persistent ARDS also argues against the notion that RIARDS is an ARDS “mimic” predominantly reflective of cardiogenic pulmonary edema. Radiographic assessment of the extent of opacities described per chest X-ray quadrant has been used as a tool to quantify the degree of pulmonary edema and ARDS severity [37]. Our analysis did not find differences in the number of chest X-ray quadrants involved in patients with RIARDS compared to persistent disease, potentially suggesting again that there are no differences in the extent of pulmonary edema related to lung injury between these groups.

Others have argued that RIARDS is essentially mild ARDS (defined by a PaO_2_: FiO_2_ > 200) [1]. This hypothesis was supported by previous findings (corroborated in our study) showing that patients with RIARDS more commonly have higher PaO_2_: FiO_2_ at time of screening [1]. However, our study and others demonstrate that RIARDS can also be seen in patients with moderate and severe ARDS [1]. We identified seven patients who had severe ARDS (defined by a PaO_2_: FiO_2_ equal to or less than 100) at time of screening and met criteria for RIARDS. The significantly higher plasma levels of inflammatory biomarkers among those with severe persistent ARDS compared to severe RIARDS suggest the possibility that varying degrees of systemic inflammation (rather than hypoxia severity at time of diagnosis) may significantly influence the clinical course.

Overall, our biological data suggest that RIARDS is associated with a less severe systemic inflammatory process compared to persistent ARDS. Plasma levels of inflammatory cytokines, namely interleukin (IL) 6 and 8, were significantly lower among those with RIARDS compared to persistent ARDS. Patients with RIARDS also had lower plasma levels of plasminogen activator inhibitor-1 (PAI-1), an anti-fibrinolytic, which expression has been previously associated with increased illness severity in ARDS [26, 38–40]. Plasma concentrations of other markers of inflammation differed between severe RIARDS and severe persistent ARDS in our sensitivity analysis focusing on severe ARDS, suggesting that the degree of inflammation severity (rather than the degree of hypoxemia at time of diagnosis) may determine resolution of ARDS. This observation is supported by results reported by Sathe et al. [41] which similarly showed that higher plasma concentration of inflammatory markers (including IL-6 and IL-8) is associated with persistent hypoxemic respiratory failure (including those not meeting ARDS criteria) compared to those with fast resolution of hypoxia.

Further studies measuring sequential levels of plasma biomarkers would be helpful to elucidate the role of inflammation in the resolution of ARDS. The differences in inflammatory markers between RIARDS and persistent disease suggest these markers could be used for early identification of RIARDS using predictive models that also include other markers of disease severity. Identifying patients with RIARDS early could help personalize their treatments and spare them from aggressive therapies. Early identification of these patients could also improve the prognostic and predictive enrichment of clinical trials, which have historically been limited by the heterogeneity of ARDS.

We confirmed our hypothesis that RIARDS would be associated with the hypoinflammatory ARDS phenotype. Notably, the differences in biomarker levels between RIARDS and persistent ARDS found in this study resemble those previously described in latent ARDS phenotypes (with lower concentrations of IL-8, IL-6, and PAI-1 among those classified as hypo- versus hyperinflammatory) [3]. Clinical similarities between RIARDS and the hypoinflammatory phenotype were also appreciated. For example, vasopressor-dependent shock and higher mortality were less common among both RIARDS and the hypoinflammatory phenotype compared to persistent ARDS and hyperinflammatory phenotype, respectively. Moreover, cirrhosis—which has previously been associated with elevated inflammatory biomarkers and is more common in the hyperinflammatory ARDS subphenotype—was more commonly observed in patients with persistent ARDS [3, 16, 42]. Taken together, these findings support the hypothesis that variation in systemic inflammation may affect the development of persistent ARDS compared to RIARDS.

Other novel clinical differences were found between RIARDS and persistent ARDS. First, RIARDS was predominantly observed in patients identified as non-Hispanic. These differences may be related to the relatively low proportion of Hispanic patients in our cohort (12% of patients), but future studies should explore the correlation between ethnicity and RIARDS. Second, a diagnosis of bacteremia was more common among persistent ARDS, possibly contributing to the higher systemic inflammatory response in these cases.

This study has several strengths. To our knowledge, it is the first study to provide simultaneous clinical and biological characterization of RIARDS in a non-randomized controlled trial population. Our cohort has detailed clinical phenotyping by board-certifying physicians in a diverse cohort. It is also the first comparison of RIARDS and persistent ARDS with the hyper- and hypoinflammatory phenotypes. The study also has some limitations. First, we analyzed one moderately sized cohort from two institutions within the same city. Larger multicenter studies and/or a validation cohort are needed to confirm these findings. Second, available plasma biomarkers were obtained at a single point in time. Serial measures may provide more information about progression and resolution of RIARDS compared with persistent disease. Third, we lack histopathologic samples which could provide more definite evidence of differences in the pathophysiology of RIARDS compared to persistent ARDS. Fourth, we studied patients with ARDS who were endotracheally intubated; further studies on the prevalence of RIARDS in patients who meet the new extended definition of ARDS (including those receiving oxygen supplementation via high flow nasal cannula) are needed. Fifth, patients with ARDS secondary to trauma were excluded from this cohort given that one of the enrollment sites is not designated a trauma center. Exclusions of these patients could potentially explain differences in RIARDS prevalence compared to previous studies. Finally, these patients were enrolled prior to the COVID-19 pandemic, so relevance to patients with COVID-19 is uncertain.

## Conclusions

We report the novel finding that RIARDS is characterized by lower concentrations of plasma biomarkers of inflammation compared to persistent ARDS, and that the hypoinflammatory ARDS phenotype is more prevalent among patients with RIARDS. Future studies are needed to explore the predictive value of the clinical and biological data presented herein for feasible identification of RIARDS in the intensive care unit, which could help personalize ARDS treatments, and improve the prognostic and predictive enrichment of clinical trials.

### Supplementary Information

**Additional file 1. Figure E1**. Study Design. All patients were enrolled in the Early Assessment of Renal and Lung Injury (EARLI) cohort from November 2008 to May 2018. We analyzed data from 215 patients who met Berlin criteria for ARDS on day 1 or 2 of the study, were endotracheally intubated at the time of meeting Berlin criteria, and had plasma biomarker measurements available. Patients met criteria for rapidly improving ARDS if any of the following criteria were met: (i) Pao2:Fio2 > 300 or (ii) Spo2:Fio2 > 315 on the day following diagnosis of ARDS (day 2) or (iii) unassisted breathing by day 2 and for the next 48 hours (defined as absence of endotracheal intubate on day 2 through day 4). **Table E1**. Comorbidities were compared in patients with RIARDS versus persistent ARDS. Cirrhosis was more commonly identified in persistent ARDS. Other comorbidities were not significantly different between each group. **Table E2**. Concomitant medical conditions were compared in patients with RIARDS versus persistent ARDS. Hypertensive crisis at time of enrollment was more common in patients with RIARDS compared to those with persistent ARDS. **Table E3**. Type of steroids administered over the first 48 hours of ARDS diagnosis in patients with RIARDS compared to those with persistent ARDS. **Table E4**. Sensitivity analysis focused on patients with severe ARDS (defined by a PaO2:FiO2 equal to or less than 100 at time of enrollment). Vasopressor-dependent shock was more commonly seen in patients with severe persistent ARDS compared to severe RIARDS. Hospital mortality was significantly higher while ICU-free days was lower in those with severe persistent ARDS compared to severe RIARDS. **Table E5**. Sensitivity analysis focused on patients with severe ARDS. Patient comorbidities did not differ significantly between RIARDS and persistent disease among those with severe ARDS. **Table E6**. Sensitivity analysis focused on patients with severe ARDS. Concomitant medical conditions did not differ significantly between RIARDS and persistent disease among those with severe ARDS. **Table E7**. Sensitivity analysis focused on patients with severe ARDS. Ventilatory parameters did not differ significantly between RIARDS and persistent disease among those with severe ARDS. **Table E8**. Sensitivity analysis focused on patients with severe ARDS (defined by a PaO2:FiO2 equal to or less than 100 at time of enrollment). Plasma inflammatory biomarkers were significantly higher in those with severe persistent ARDS compared to severe RIARDS. **Table E9**. Sensitivity analysis comparing RIARDS and persistent disease among cases allocated to the hyperinflammatory phenotype. Similar to the results seen in the overall cohort, compared to patients with hyperinflammatory persistent ARDS, patients with hyperinflammatory RIARDS had significantly lower in-hospital mortality at 28 days and higher ICU-free days. However, contrary to results seen in the overall cohort, vasopressor-dependent shock on day 1 was equally prevalent. Severe hypoxemia was more commonly seen in persistent ARDS and not appreciated in RIARDS. **Table E10**. Sensitivity analysis comparing RIARDS and persistent disease among cases allocated to the hyperinflammatory phenotype. Microbiology and medications received did not differ between each group. **Table E11**. Sensitivity analysis comparing RIARDS and persistent disease among cases allocated to the hyperinflammatory phenotype. No significant differences were found in plasma inflammatory biomarker concentration between patients with RIARDS and persistent ARDS. **Table E12**. Total counts of missing values. Ventilatory parameters stratified by persistent ARDS versus RIARDS. **Table E13**. Total counts of missing values. Biomarkers stratified by persistent ARDS versus RIARDS. **Table E14**. Total counts of missing values. Comorbidities and concomitant medical conditions stratified by persistent ARDS versus RIARDS.

**Additional file 2. Supplementary Methods**. This section provides additional information regarding enrollment of participants in our cohort and review of clinical charts. We also provide details on the collection and processing of plasma biomarkers. Finally, we explain how we calculated ICU-free days, ventilator-free days and hospital mortality.

**Additional file 3. Visual Abstract**. Rapidly improving acute respiratory distress syndrome (RIARDS) is a subgroup of ARDS in which hypoxemia significantly improves within 24 hours after initiation of mechanical ventilation. We analyzed data in patients with RIARDS (defined as (i) PaO2:FiO2>300 or (ii) SpO2:FiO2>315 on the day following diagnosis of ARDS or (iii) unassisted breathing by day 2 and for the next 48 hours) and in patients with persistent ARDS. Patients with RIARDS had better clinical outcomes compared to those with persistent ARDS, with lower hospital mortality and more ICU-free days. Plasma levels of inflammatory markers were significantly lower among patients with RIARDS. The hypoinflammatory phenotype of ARDS was more common among patients with RIARDS.

## Data Availability

The datasets used and analyzed during the current study are available from the corresponding author on reasonable request.

## Abbreviations

ARDS: Acute respiratory distress syndrome
RIARDS: Rapidly improving acute respiratory distress syndrome
EARLI: The Early Assessment of Renal and Lung Injury
IL-8: Interleukin-8
IL-6: Interleukin-6
TNF alpha: Tumor necrosis factor alpha
IL-10: Interleukin-10
IFN gamma: Interferon gamma
IL-1Beta: Interleukin-1 beta
CX3CL1: Gene encoding chemokine ligand 1
sTNFR-1: Soluble tumor necrosis factor receptor-1
sRAGE: Soluble receptor for advanced glycation end-products
Ang-2: Angiopoietin-2
ICAM-1: Intercellular adhesion molecule 1
PAI-1: Plasminogen activator inhibitor 1.
PIP: Peak inspiratory pressure
FiO_2_: Fraction of inspired oxygen
PEEP: Positive end-expiratory pressure
paCO_2_: Partial pressure of carbon dioxide in arterial blood gas
MAP: Mean arterial pressure
SBP: Systolic blood pressure
