# Supplementary material for: Rapidly improving ARDS differs clinically and biologically from persistent ARDS
Source: Crit Care. 2024 Apr 22;28:132. doi: 10.1186/s13054-024-04883-6 (PMC11034037; doi:10.1186/s13054-024-04883-6)
Supplement: Supplementary file 1 — Additional file 1. Figure E1. Study Design. All patients were enrolled in the Early Assessment of Renal and Lung Injury (EARLI) cohort from November 2008 to May 2018. We analyzed data from 215 patients who met Berlin criteria for ARDS on day 1 or 2 of the study, were endotracheally intubated at the time of meeting Berlin criteria, and had plasma biomarker measurements available. Patients met criteria for rapidly improving ARDS if any of the following criteria were met: (i) Pao2:Fio2 > 300 or (ii) Spo2:Fio2 > 315 on the day following diagnosis of ARDS (day 2) or (iii) unassisted breathing by day 2 and for the next 48 hours (defined as absence of endotracheal intubate on day 2 through day 4). Table E1. Comorbidities were compared in patients with RIARDS versus persistent ARDS. Cirrhosis was more commonly identified in persistent ARDS. Other comorbidities were not significantly different between each group. Table E2. Concomitant medical conditions were compared in patients with RIARDS versus persistent ARDS. Hypertensive crisis at time of enrollment was more common in patients with RIARDS compared to those with persistent ARDS. Table E3. Type of steroids administered over the first 48 hours of ARDS diagnosis in patients with RIARDS compared to those with persistent ARDS. Table E4. Sensitivity analysis focused on patients with severe ARDS (defined by a PaO2:FiO2 equal to or less than 100 at time of enrollment). Vasopressor-dependent shock was more commonly seen in patients with severe persistent ARDS compared to severe RIARDS. Hospital mortality was significantly higher while ICU-free days was lower in those with severe persistent ARDS compared to severe RIARDS. Table E5. Sensitivity analysis focused on patients with severe ARDS. Patient comorbidities did not differ significantly between RIARDS and persistent disease among those with severe ARDS. Table E6. Sensitivity analysis focused on patients with severe ARDS. Concomitant medical conditions did not differ significan [file 13054_2024_4883_MOESM1_ESM.docx]

**Online Data Supplement - FIGURES**

**Rapidly Improving ARDS Differs Clinically and Biologically From Persistent ARDS**

**Authors**

Patricia L Valda Toro MD MS^1^, Andrew Willmore ^2^, Nelson E Wu MPH^2^, Kevin L Delucchi PhD^4^, Alejandra Jauregui^2^, Pratik Sinha MD PhD^7^, Kathleen D Liu MD PhD^2,3^, Carolyn M Hendrickson MD MPH^2,3^, Aartik Sarma MD^2^, Lucile P A Neyton PhD^2^, Aleksandra Leligdowicz MD PhD^8^, Charles R Langelier MD PhD^5, 6^, Hanjing Zhuo MPH^2^, Chayse Jones^2^, Kirsten N Kangelaris MD^9^, Antonio D Gomez MD^2,3^, Michael A Matthay MD^2,3^, Carolyn S Calfee MD MAS^2,3^

**Corresponding autor:** Patricia L. Valda Toro MD MS
 [patricia.valdatoro@pennmedicine.upenn.edu](mailto:patricia.valdatoro@pennmedicine.upenn.edu)

**Supplement Figure E1: Study design**

**
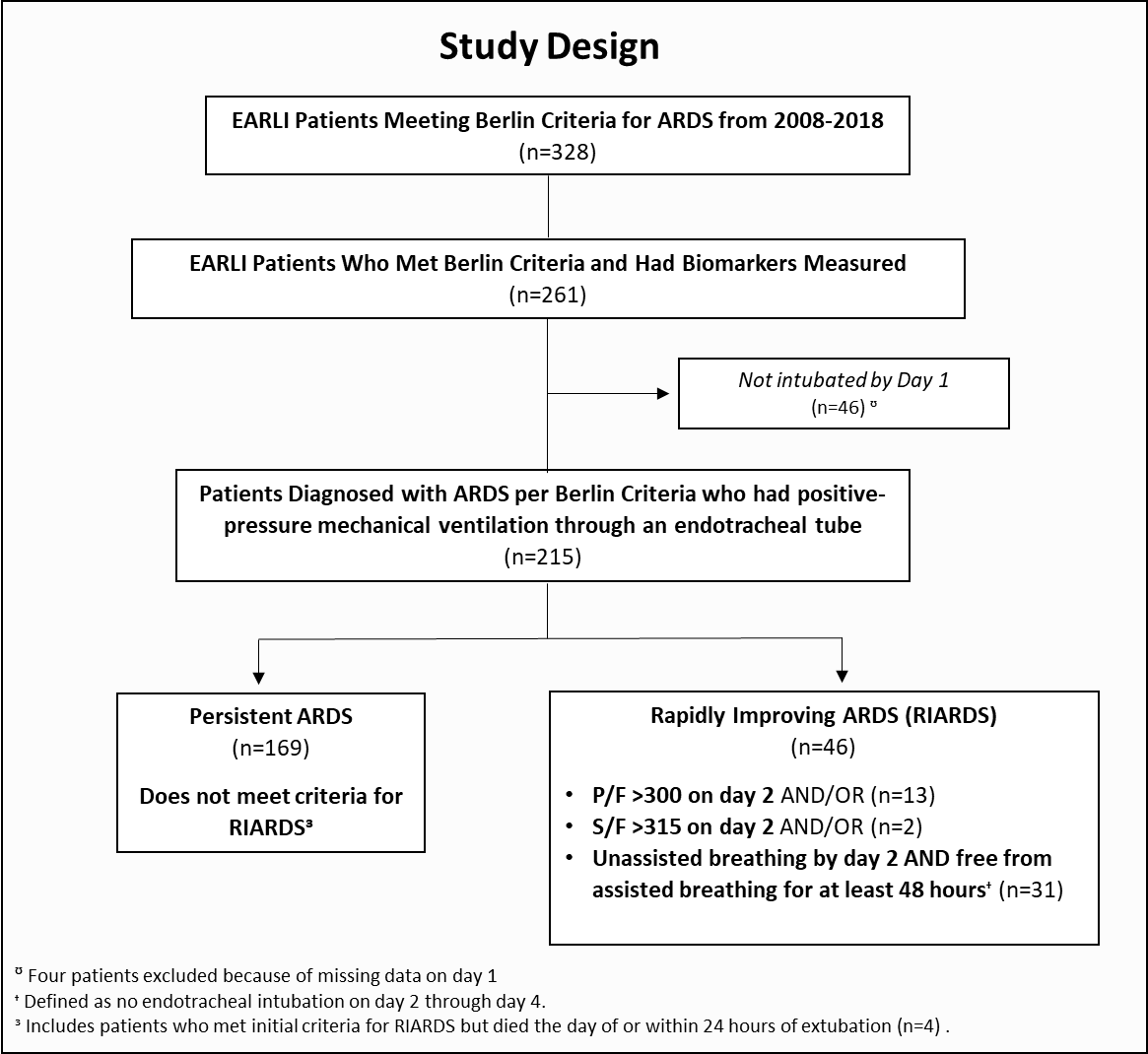
**

**Table E1: Patient comorbidities in patients stratified by presence or absence of RIARDS.**

| Patient Comorbidities | | | |
| --- | --- | --- | --- |
|  | **Persistent ARDS**  N=169 | **RIARDS**  N= 46 | **p-value** |
| Cirrhosis, n (%) | **21 (12)** | **1 (2)** | **0.05** |
| Acquired Immunodeficiency Syndrome, n (%) | 12 (7) | 0 (0) | 0.07 |
| Obstructive Sleep Apnea, n (%) | 12 (7) | 0 (0) | 0.07 |
| Human Immunodeficiency Virus, n (%) | 18 (11) | 1 (2) | 0.14 |
| End Stage Renal Disease, n (%) | 14 (8) | 7 (15) | 0.16* |
| Other immunosuppressive state, n (%)^§^ | 107 (63) | 24 (52) | 0.18* |
| Coronary Artery Disease, n (%) | 40 (24) | 15 (33) | 0.22* |
| Interstitial Lung Disease, n (%) | 3 (2) | 2 (4) | 0.29 |
| Chronic Kidney Disease, n (%) | 42 (25) | 15 (33) | 0.29* |
| Chronic Obstructive Pulmonary Disease, n (%) | 45 (27) | 9 (20) | 0.33* |
| Diastolic dysfunction, n (%)^‡^ | 61 (36) | 15 (33) | 0.34* |
| Hypertension, n (%) | 91 (54) | 28 (61) | 0.40* |
| Multiple Myeloma, n (%) | 3 (2) | 2 (4) | 0.44 |
| Leukemia, n (%) | 6 (4) | 0 (0) | 0.49 |
| Alcohol Use, n (%) | 61 (36) | 17 (37) | 0.50* |
| Left ventricular dysfunction, n (%)^‡^ | 59 (35) | 15 (33) | 0.58* |
| Restrictive Lung Disease, n (%) | 4 (2) | 2 (4) | 0.61 |
| Solid Tumor, n (%) | 31 (18) | 7 (15) | 0.62* |
| On immunosuppressive drugs, n (%) | 25 (15) | 5 (11) | 0.63 |
| Right ventricular dysfunction, n (%)^‡^ | 51 (30) | 13 (28) | 0.63* |
| Cerebrovascular Disease, n (%) | 30 (18) | 7 (15) | 0.69* |
| Asthma, n (%) | 13 (8) | 2 (4) | 0.74 |
| Solid Organ Transplant, n (%) | 6 (4) | 2 (4) | 0.75 |
| Home Oxygen, n (%) | 19 (11) | 4 (9) | 0.79 |
| Outpatient Dialysis, n (%) | 13 (8) | 4 (9) | 0.86 |
| Diabetes, n (%) | 55 (33) | 15 (33) | 0.99* |
| Tobacco Smoking, n (%) | 81 (48) | 22 (48) | 0.99* |
| Lung Transplant, n (%) | 2 (1) | 0 (0) | 1.00 |
| Lymphoma, n (%) | 3 (2) | 0 (0) | 1.00 |
| Lung Cancer, n (%) | 7 (4) | 1 (2) | 1.00 |
| Pancreatitis, n (%) | 7 (4) | 1 (2) | 1.00 |
| Thyroid Disease, n (%) | 21 (13) | 5 (11) | 1.00 |
| Fisher’s test was used to determine statistical significance unless otherwise specified. * Pearson’s chi-square test was used to determine statistical significance. ‡ Values are missing for several patients as outlined in supplement table ten.  § Includes immunosuppression from HIV positivity, AIDS, diabetes, cirrhosis, malignancy (solid or liquid), immunodeficiencies (i.e. common variable immunodeficiency), and use immunosuppressive medications. | | | |

**Table E2: Concomitant medical conditions at time of ARDS diagnosis in patients stratified by presence or absence of RIARDS.**

| Concomitant Medical Conditions | | | |
| --- | --- | --- | --- |
|  | **Persistent ARDS**  N=169 | **RIARDS**  N= 46 | **p-value** |
| Hypertensive Crisis, n (%) | **5 (3)** | **6 (13)** | **0.01** |
| Acute Renal Failure, n (%) | 110 (65) | 29 (63) | 0.80* |
| Acute Renal Failure Requiring Renal Replacement Therapy, n (%) ^‡^ | 35 (21) | 10 (22) | 0.82* |
| Volume overload, n (%) | 57 (34) | 16 (35) | 0.89* |
| Fisher’s test was used to determine statistical significance unless otherwise specified. * Pearson’s chi-square test was used to determine statistical significance.  ‡ Values are missing for several patients as outlined in supplement table ten. | | | |

**Table E3: Type of steroids administered within 48 hours of ARDS diagnosis in patients stratified by presence or absence of RIARDS.**

| **Type of Steroids Given Within 48 hours of ARDS diagnosis** | | | |
| --- | --- | --- | --- |
|  | **Persistent ARDS** | **RIARDS** | **Total** |
| **Day 1** | **N=169** | **N=46** | **N=215** |
| Methylprednisolone, n (%) | 16 (9) | 2 (4) | 18 (8) |
| Hydrocortisone, n (%) | 29 (17) | 3 (7) | 32 (15) |
| Dexamethasone, n (%) | 1 (0.6) | 0 (0) | 1 (0.5) |
| Prednisone, n (%) | 1 (0.6) | 1 (2) | 2 (1) |
| Dexamethasone and hydrocortisone, n (%) | 2 (1) | 0 (0) | 2 (1) |
| Prednisone and hydrocortisone, n (%) | 1 (0.6) | 0 (0) | 1 (0.5) |
| **Day 2** | **N=169** | **N=46** | **N=215** |
| Methylprednisolone, n (%) | 20 (12) | 3 (7) | 23 (11) |
| Hydrocortisone, n (%) | 30 (18) | 1 (2) | 31 (14) |
| Dexamethasone, n (%) | 1 (0.6) | 0 (0) | 1 (0.5) |
| Prednisone, n (%) | 2 (1) | 0 (0) | 2 (1) |
| Methylprednisolone and hydrocortisone, n (%) | 2 (1) | 0 (0) | 2 (1) |
| Prednisone, hydrocortisone, methylprednisolone, n (%) | 1 (0.6) | 0 (0) | 1 (0.5) |

**Table E4: Clinical characteristics in patients with severe ARDS at time of enrollment.**

| General Characteristics for Patients with Severe ARDS Only | | | |
| --- | --- | --- | --- |
|  | **Persistent ARDS**  (N =79) | **RIARDS**  (N=7) | **p-value** |
| Age, median (IQR) | 65 (50, 79) | 47 (25, 87) | 0.67 |
| BMI, median (IQR) | 25 (22, 30) | 23 (22, 28) | 0.30 |
| Gender |  |  |  |
| Male, n (%) | 49 (62) | 6 (86) | 0.46 |
| Female, n (%) | 29 (37) | 1 (14) |  |
| Transgender, n (%) | 1 (1) | 0 (0) |  |
| Race |  |  |  |
| Caucasian, n (%) | 36 (46) | 6 (86) | 0.24 |
| African American, n (%) | 9 (11) | 1 (14) |  |
| Asian, n (%) | 22 (28) | 0 (0) |  |
| Pacific Islander, n (%) | 0 (0) | 0 (0) |  |
| Native American, n (%) | 0 (0) | 0 (0) |  |
| Other, n (%) | 11 (14) | 0 (0) |  |
| Unknown, n (%) | 1 (1) | 0 (0) |  |
| Ethnicity |  |  |  |
| Hispanic, n (%) | 14 (18) | 0 (0) | 0.63 |
| Non-Hispanic, n (%) | 64 (81) | 7 (100) |  |
| Primary ARDS Risk Factor |  |  |  |
| Pneumonia, n (%) | 28 (35) | 1 (14) | 0.35 |
| Sepsis, n (%) | 31 (39) | 2 (29) |  |
| Aspiration, n (%) | 14 (18) | 4 (57) |  |
| Transfusion, n (%) | 3 (4) | 0 (0) |  |
| Drug Overdose, n (%) | 1 (1) | 0 (0) |  |
| Other, n (%) | 1 (1) | 0 (0) |  |
| None, n (%) | 1 (1) | 0 (0) |  |
| “Pure” ARDS (non-cardiogenic edema) vs. mixed cardiogenic and non-cardiogenic edema† | | | |
| “Pure” ARDS, n (%) | 59 (75) | 5 (71) | 1.0 |
| Mixed etiology of pulmonary edema, n (%) | 20 (25) | 2 (29) |  |
| CXR Quadrants Involved, Qualifying CXR |  |  |  |
| Two, n (%) | 14 (18) | 4 (57) | 0.15 |
| Three, n (%) | 24 (30) | 1 (14) |  |
| Four, n (%) | 37 (47) | 2 (29) |  |
| Unknown | 4 (5) | 0 (0) |  |
| Severity of Disease within 1 day of ARDS diagnosis | |  |  |
| APACHE III score, median (IQR) | 145 (116, 175) | 133 (81, 141) | 0.15 |
| Vasopressor-dependent shock, n (%) | 68 (86) | 3 (43) | **0.02** |
| RRT-requiring renal failure, n (%) | 19 (24) | 1 (14) | 1.0 |
| Clinical Outcomes |  |  |  |
| Hospital Mortality at 28 days, n (%) | 49 (62) | 1 (14) | **0.02** |
| ICU-free days, median (IQR) | 0 (0, 13) | 21 (21, 22) | **<0.001** |
| Ventilator-free days, median (IQR) | 0 (0, 19) | 26 (25, 26) | **<0.001** |
| Mann Whitney U Test or Fisher’s test were used to determine statistical significance.  †Determined by two board-certified physician who independently reviewed radiological and clinical data. | | | |

**Table E5: Patient comorbidities in patients with severe ARDS at time of enrollment.**

| Patient Comorbidities  Patients with Severe ARDS Only | | | |
| --- | --- | --- | --- |
|  | **Persistent ARDS**  (N =79) | **RIARDS**  (N=7) | **p-value** |
| Interstitial Lung Disease, n (%) | 0 (0) | 1 (14) | 0.08 |
| Restrictive Lung Disease, n (%) | 1 (1) | 1 (14) | 0.16 |
| Right ventricular dysfunction, n (%)^‡^ | 28 (35) | 1 (14) | 0.19 |
| Other immunosuppressive state, n (%)^§^ | 44 (56) | 2 (29) | 0.24 |
| Diastolic dysfunction, n (%)^‡^ | 25 (32) | 3 (43) | 0.25 |
| COPD, n (%) | 18 (23) | 0 (0) | 0.34 |
| Left ventricular dysfunction, n (%)^‡^ | 29 (37) | 2 (29) | 0.40 |
| End Stage Renal Disease, n (%) | 7 (9) | 1 (14) | 0.51 |
| Outpatient Dialysis, n (%) | 6 (8) | 1(14) | 0.51 |
| Cirrhosis, n (%) | 12 (15) | 0 (0) | 0.59 |
| Solid Tumor, n (%) | 11 (14) | 0 (0) | 0.59 |
| Diabetes, n (%) | 21 (27) | 1 (14) | 0.67 |
| Tobacco Smoking, n (%) | 34 (43) | 2 (29) | 0.69 |
| Acquired Immunodeficiency Syndrome, n (%) | 4 (5) | 0 (0) | 1.00 |
| Obstructive Sleep Apnea, n (%) | 7 (9) | 0 (0) | 1.00 |
| Human Immunodeficiency Virus, n (%) | 7 (9) | 0 (0) | 1.00 |
| Coronary Artery Disease, n (%) | 18 (23) | 1 (14) | 1.00 |
| Multiple Myeloma, n (%) | 1 (1) | 0 (0) | 1.00 |
| Hypertension, n (%) | 40 (51) | 3 (43) | 1.00 |
| Cerebrovascular Disease, n (%) | 12 (15) | 1 (14) | 1.00 |
| Asthma, n (%) | 6 (8) | 0 (0) | 1.00 |
| Leukemia, n (%) | 5 (6) | 0 (0) | 1.00 |
| Lymphoma, n (%) | 1 (1) | 0 (0) | 1.00 |
| Lung Cancer, n (%) | 3 (4) | 0 (0) | 1.00 |
| Pancreatitis, n (%) | 2 (3) | 0 (0) | 1.00 |
| Chronic Kidney Disease, n (%) | 13 (16) | 1 (14) | 1.00 |
| Home Oxygen, n (%) | 7 (9) | 0 (0) | 1.00 |
| Thyroid Disease, n (%) | 10 (13) | 0 (0) | 1.00 |
| On immunosuppressive drugs, n (%) | 9 (11) | 0 (0) | 1.00 |
| Alcohol Use, n (%) | 33 (42) | 3 (43) | 1.00 |
| Lung Transplant, n (%) | 0 (0) | 0 (0) | - |
| Solid Organ Transplant, n (%) | 0 (0) | 0 (0) | - |
| Fisher’s test was used to determine statistical significance. ‡ Values are missing for several patients as outlined in supplement table ten.  § Includes immunosuppression from HIV positivity, AIDS, diabetes, cirrhosis, malignancy (solid or liquid), immunodeficiencies (i.e. common variable immunodeficiency), and use immunosuppressive medications. | | | |

**Table E6: Concomitant medical conditions in patients with severe ARDS at time of enrollment.**

| Concomitant Medical Conditions  Patients with Severe ARDS Only | | | | |
| --- | --- | --- | --- | --- |
|  | **Persistent ARDS**  (N =79) | **RIARDS**  (N=7) | | **p-value** |
| Acute Renal Failure Requiring Renal Replacement Therapy, n (%)^‡^ | 19 (24) | 1 (14) | 0.33 | |
| Acute Renal Failure, n (%) | 56 (71) | 6 (86) | 0.67 | |
| Volume overload, n (%) | 28 (35) | 3 (43) | 0.70 | |
| Hypertensive Crisis, n (%) | 1 (1) | 0 (0) | 1.00 | |
| Fisher’s test was used to determine statistical significance.  ‡ Values are missing for several patients as outlined in supplement table ten. | | | | |

**Table E7: Ventilatory parameters in patients with severe ARDS at time of enrollment.**

| Ventilatory Parameters  Patients with Severe ARDS ONLY | | | |
| --- | --- | --- | --- |
|  | **Persistent RIARDS**  N=79 | **RIARDS**  N= 7 | **p-value** |
| PIP, median (IQR)^‡^ | 32 (27, 38) | 23 (17, 37) | 0.06 |
| PEEP, median (IQR) | 8 (5, 10) | 5 (5, 10) | 0.26 |
| paCO2, median (IQR) | 43 (33, 58) | 49 (44, 52) | 0.32 |
| FiO2, median (IQR)^‡^ | 1 (0.8, 1) | 1 (0.5, 1) | 0.36 |
| Plateau Pressure, median (IQR)^‡^ | 25 (22, 29) | 24 (21, 27) | 0.58 |
| Mann Whitney U Test were used to determine statistical significance  ‡ Values are missing for several patients as outlined in supplement table eight. | | | |
| PIP: peak inspiratory pressure; FiO2: fraction of inspired oxygen; PEEP: Positive end-expiratory pressure; paCO2: partial pressure of carbon dioxide in arterial blood gas. | | | |

**Table E8: Plasma biomarker levels in patients with severe ARDS at time of enrollment.**

| Biomarkers in RIARDS versus Non-RIARDS  Patients with Severe ARDS ONLY | | | | | | | |
| --- | --- | --- | --- | --- | --- | --- | --- |
|  | **Persistent ARDS**  N=79  Median (IQR) | **RIARDS**  N= 7  Median (IQR) | **p-value** | | | **p-value, adjusted^‡^** | |
| INFLAMMATION MEDIATORS | | | | | | | |
| IL-10 (pg/ml) | 51 (31, 294) | 22 (21, 29) | **0.001** | | | | **0.01** |
| IL-8 (pg/ml) | 246 (48, 3279) | 14 (10, 16) | **0.001** | | | | **0.01** |
| TNF alpha (pg/ml) | 108 (70, 198) | 60 (53, 72) | **0.005** | | | | 0.06 |
| IL-6 (pg/ml) | 2141 (133, 10326) | 51 (6, 494) | **0.006** | | | | 0.08 |
| IL-1Beta (pg/ml) | 88 (67, 119) | 58 (53, 81) | **0.009** | | | | 0.11 |
| IFN gamma (pg/ml) | 119 (95, 152) | 82 (69, 107) | **0.009** | | | | 0.12 |
| CX3CL1 (pg/ml) | 13814 (11488, 16133) | 11029 (8860, 13386) | **0.04** | | | | 0.55 |
| sTNFR-1 (pg/mL) | 6851 (2465, 11634) | 5287 (1359, 6047) | 0.23 | | | | 1 |
| LUNG INJURY | | | | | | | |
| Alveolar type I | | | | | | | |
| sRAGE (pg/ml) | 5368 (2886, 9159) | 4265 (787, 6520) | | 0.44 | | | 1 |
| Endothelial Cell | | | | | | | |
| Ang-2 (pg/ml) | 10182 (5842, 18355) | 4886 (2051, 12364) | | | **0.04** | | 0.53 |
| ICAM-1 (pg/mL) | 789943 (307630, 1679860) | 455517 (344262, 771210) | | | 0.24 | | 1 |
| COAGULATION | | | | | | | |
| Protein C (% control) | 62 (24, 114) | 143 (91, 205) | | | **0.006** | | 0.08 |
| PAI-1 (ng/mL) | 25 (8, 62) | 5 (4, 11) | | | **0.007** | | 0.09 |
| ‡ Bonferroni adjusted p-value. Mann Whitney U Test was used to calculate uncorrected alpha. | | | | | | | |
| IL-8: Interleukin-8; IL-6: Interleukin-6; TNF alpha: tumor necrosis factor alpha; IL-10: Interleukin-10; IFN gamma: interferon gamma; IL-1Beta: interleukin-1 Beta; CX3CL1: gene encoding chemokine ligand 1; sTNFR-1: soluble tumor necrosis factor receptor-1; sRAGE: soluble receptor for advanced glycation end-products; Ang-2: angiopoietin-2; ICAM-1: intercellular adhesion molecule 1; PAI-1: plasminogen activator inhibitor 1. | | | | | | | |

**Table E9: Clinical characteristics in patients with hyperinflammatory ARDS.**

| General Characteristics for Patients with Hyperinflammatory ARDS Only | | | |
| --- | --- | --- | --- |
|  | **Persistent ARDS**  (N =82) | **RIARDS**  (N=10) | **p-value** |
| Age, median (IQR) | 66 (56, 79) | 66 (61, 85) | 0.70 |
| BMI, median (IQR) | 23 (21, 25) | 26 (24, 31) | **0.05** |
| Gender |  |  |  |
| Male, n (%) | 51 (62) | 4 (40) | 0.19 |
| Female, n (%) | 31 (38) | 6 (60) |  |
| Race |  |  |  |
| Caucasian, n (%) | 33 (40) | 5 (50) | 0.96 |
| African American, n (%) | 8 (10) | 1 (10) |  |
| Asian, n (%) | 28 (34) | 3 (30) |  |
| Pacific Islander, n (%) | 0 (0) | 0 (0) |  |
| Native American, n (%) | 0 (0) | 0 (0) |  |
| Other, n (%) | 12 (15) | 1 (10) |  |
| Unknown, n (%) | 1 (1) | 0 (0) |  |
| Ethnicity |  |  |  |
| Hispanic, n (%) | 13 (16) | 0 (0) | 0.42 |
| Non-Hispanic, n (%) | 68 (83) | 10 (100) |  |
| Unknown, n (%) | 1 (1) | 0 (0) |  |
| Hospital Setting |  |  |  |
| Tertiary Care Center, n (%) | 60 (73) | 9 (90) | 0.44 |
| Urban Safety Net Hospital, n (%) | 22 (27) | 1 (10) |  |
| Primary ARDS Risk Factor |  |  |  |
| Pneumonia, n (%) | 25 (30) | 2 (20) | 0.72 |
| Sepsis, n (%) | 40 (49) | 6 (60) |  |
| Aspiration, n (%) | 11 (13) | 1 (10) |  |
| Transfusion, n (%) | 3 (4) | 1 (10) |  |
| Drug Overdose, n (%) | 1 (1) | 0 (0) |  |
| Other, n (%) | 1 (1) | 0 (0) |  |
| None, n (%) | 1 (1) | 0 (0) |  |
| Severity of ARDS at enrollment by PaO2/FiO2 |  |  |  |
| Mild, n (%) | 8 (10) | 6 (60) | **<0.001** |
| Moderate, n (%) | 25 (30) | 4 (40) |  |
| Severe, n (%) | 49 (60) | 0 (0) |  |
| “Pure” ARDS (non-cardiogenic edema) vs. mixed cardiogenic and non-cardiogenic edema† | | | |
| “Pure” ARDS, n (%) | 58 (71) | 8 (80) | 0.72 |
| Mixed etiology of pulmonary edema, n (%) | 24 (29) | 2 (20) |  |
| CXR Quadrants Involved, Qualifying CXR |  |  |  |
| Two, n (%) | 21 (26) | 3 (30) | 0.27 |
| Three, n (%) | 22 (27) | 4 (40) |  |
| Four, n (%) | 36 (44) | 2 (20) |  |
| Unknown | 3 (4) | 1 (10) |  |
| Severity of Disease within 1 day of ARDS diagnosis | |  |  |
| APACHE III score, median (IQR) | 153 (129, 183) | 110 (86, 162) | **0.02** |
| Vasopressor-dependent shock, n (%) | 81 (99) | 10 (100) | 1.0 |
| RRT-requiring renal failure, n (%) | 25 (30) | 0 (0) | **0.03** |
| Clinical Outcomes |  |  |  |
| Hospital Mortality at 28 days, n (%) | 56 (68) | 2 (20) | **0.01** |
| ICU-free days, median (IQR) | 0 (0, 12) | 24 (20, 25) | **<0.001** |
| Ventilator-free days, median (IQR) | 0 (0, 15) | 26 (25, 26) | **<0.001** |
| Mann Whitney U Test or Fisher’s test were used to determine statistical significance.  †Determined by two board-certified physician who independently reviewed radiological and clinical data. | | | |

**Table E10: Clinical parameters in patients with hyperinflammatory ARDS.**

| Clinical Parameters | | | | |
| --- | --- | --- | --- | --- |
|  | | **Persistent ARDS**  (N =82) | **RIARDS**  (N=10) | **p-value** |
| Ventilatory Parameters on Day 1 of ARDS diagnosis | | | | |
| FiO2 (%), median (IQR) † | 1 (0.7, 1) | | 0.6 (0.5, 0.8) | **0.03** |
| PEEP (cmH_2_O), median (IQR) | 5 (5, 10) | | 5 (5, 5) | 0.07 |
| Plateau Pressure (cmH_2_O), median (IQR)† | 22 (19, 27) | | 19 (17, 23) | 0.09 |
| paCO2 (mmHg), median (IQR) | 35 (31, 45) | | 33 (30, 45) | 0.62 |
| PIP (cmH_2_O), median (IQR)† | 30 (24, 36) | | 27 (25, 33) | 0.63 |
| Arterial Pressure on Day 1 of ARDS diagnosis | | | | |
| MAP (mmHg), median (IQR) | 74 (64, 87) | | 76 (66, 90) | 0.88 |
| SBP (mmHg), median (IQR) | 109 (91, 124) | | 110 (96, 126) | 0.64 |
| Medications Received Within 48 hours of ARDS diagnosis | | | | |
| Steroids, n (%) | 42 (51) | | 2 (20) | 0.09 |
| Diuretics, n (%) | 7 (9) | | 1 (10) | 1.00 |
| Antibiotics, n (%) | 77 (94) | | 10 (100) | 1.00 |
| Antivirals, n (%) | 23 (28) | | 1 (10) | 0.45 |
| Antifungals, n (%) | 13 (16) | | 0 (0) | 0.35 |
| Microbiology Within 48 hours of ARDS diagnosis | | | | |
| Pulmonary Infection |  | |  |  |
| Bacterial, n (%) | 16 (20) | | 1 (10) | 0.68 |
| Viral, n (%) | 8 (10) | | 1 (10) | 1.00 |
| Fungal, n (%) | 0 (0) | | 0 (0) | ^-^ |
| Bacteremia, n (%) | 24 (29) | | 1 (10) | 0.28 |
|  |  | |  |  |
| PIP: peak inspiratory pressure; FiO2: fraction of inspired oxygen; PEEP: Positive end-expiratory pressure; paCO2: partial pressure of carbon dioxide in arterial blood gas; MAP: mean arterial pressure: SBP: systolic blood pressure. | | | | |
| Mann Whitney U Test or Fisher’s test were used to determine statistical significance unless otherwise specified.  *Pearson’s chi-square test was used to determine statistical significance. †Missing values for some patients as indicated in supplement table eight. | | | | |

**Table E11: Plasma biomarker levels in patients with hyperinflammatory ARDS.**

| Biomarkers in RIARDS versus Persistent ARDS  Patients with Hyperinflammatory ARDS ONLY | | | | | | | |
| --- | --- | --- | --- | --- | --- | --- | --- |
|  | **Persistent ARDS**  N =82  Median (IQR) | **RIARDS**  N= 10  Median (IQR) | **p-value** | | | **Bonferroni adjusted**  **p-value^‡^** | |
| INFLAMMATION MEDIATORS | | | | | | | |
| IL-8 (pg/ml) | 516 (130, 3780) | 149 (69, 228) | **0.02** | | | | 0.26 |
| TNF alpha (pg/ml) | 144 (101, 224) | 75 (72, 237) | 0.10 | | | | 1 |
| IL-10 (pg/ml) | 82 (41, 325) | 47 (23, 159) | 0.11 | | | | 1 |
| IL-6 (pg/ml) | 4839 (730, 13986) | 1036 (474, 5108) | 0.16 | | | | 1 |
| IL-1Beta (pg/ml) | 100 (73, 133) | 78 (67, 113) | 0.28 | | | | 1 |
| CX3CL1 (pg/ml) | 14791 (12901, 17157) | 14331 (11216, 17035) | 0.42 | | | | 1 |
| IFN gamma (pg/ml) | 129 (107, 159) | 119 (107, 156) | 0.52 | | | | 1 |
| sTNFR-1 (pg/mL) | 9829 (5192, 14355) | 7850 (4159, 17776) | 0.57 | | | | 1 |
| LUNG INJURY | | | | | | | |
| Alveolar type I | | | | | | | |
| sRAGE (pg/ml) | 5568 (3588, 9410) | 3599 (2200, 5339) | | 0.12 | | | 1 |
| Endothelial Cell | | | | | | | |
| ICAM-1 (pg/mL) | 1344573 (516363, 2027613) | 915853 (456391, 1416196) | | | 0.38 | | 1 |
| Ang-2 (pg/ml) | 13424 (7297, 26795) | 11560 (5173, 16720) | | | 0.44 | | 1 |
| COAGULATION | | | | | | | |
| Protein C (% control) | 60 (21, 107) | 62 (56, 117) | | | 0.24 | | 1 |
| PAI-1 (ng/mL) | 43 (19, 81) | 35 (15, 43) | | | 0.26 | | 1 |
| ‡ Bonferroni adjusted p-value. Mann Whitney U Test was used to calculate uncorrected alpha. | | | | | | | |
| IL-8: Interleukin-8; IL-6: Interleukin-6; TNF alpha: tumor necrosis factor alpha; IL-10: Interleukin-10; IFN gamma: interferon gamma; IL-1Beta: interleukin-1 Beta; CX3CL1: gene encoding chemokine ligand 1; sTNFR-1: soluble tumor necrosis factor receptor-1; sRAGE: soluble receptor for advanced glycation end-products; Ang-2: angiopoietin-2; ICAM-1: intercellular adhesion molecule 1; PAI-1: plasminogen activator inhibitor 1. | | | | | | | |

**Table E12: Missing values, ventilatory parameters.**

| **Count of Missing Values: Ventilatory Parameters** | | | |
| --- | --- | --- | --- |
|  | **Persistent ARDS** | **RIARDS** | **Total** |
| **All Included Cohort** | **N=169** | **N=46** | **N=215** |
| PEEP, n (%) | 6 (4) | 1 (2) | 7 (3) |
| PIP, n (%) | 15 (9) | 6 (13) | 21 (10) |
| Plateau Pressure, n (%) | 52 (31) | 12 (26) | 64 (30) |
| FiO2, n (%) | 11 (7) | 2 (4) | 13 (6) |
| paCO2, n (%) | 4 (2) | 1 (2) | 5 (2) |
| SBP, n (%) | 2 (1) | 0 (0) | 2 (1) |
| MAP, n (%) | 2 (1) | 0 (0) | 2 (1) |
| **Sensitivity Analysis (severe ARDS)** | **N=79** | **N=7** | **N=86** |
| PEEP, n (%) | 3 (4) | 0 (0) | 3 (3) |
| PIP, n (%) | 8 (10) | 0 (0) | 8 (9) |
| Plateau Pressure, n (%) | 27 (34) | 5 (71) | 32 (37) |
| FiO2, n (%) | 7 (9) | 0 (0) | 7 (8) |
| paCO2, n (%) | 2(3) | 0 (0) | 2 (2) |
| **Sensitivity Analysis (hyperinflammatory ARDS)** | **N=82** | **N=10** | **N=92** |
| PEEP, n (%) | 2 (2) | 1 (10) | 3 (3) |
| PIP, n (%) | 10 (12) | 2 (20) | 12 (13) |
| Plateau Pressure, n (%) | 29 (35) | 2 (20) | 31 (34) |
| FiO2, n (%) | 6 (7) | 1 (10) | 7 (8) |
| paCO2, n (%) | 1 (1) | 0 (0) | 1 (1) |
| SBP, n (%) | 1 (1) | 0 (0) | 1 (1) |
| MAP, n (%) | 1 (1) | 0 (0) | 1 (1) |
| **PIP**: peak inspiratory pressure; **FiO2**: fraction of inspired oxygen; **PEEP**: Positive end-expiratory pressure; **paCO2**: partial pressure of carbon dioxide in arterial blood gas; **MAP**: mean arterial pressure: **SBP**: systolic blood pressure. | | | |

**Table E13: Missing values, biomarkers.**

| **Count of Missing Values: Biomarkers** | | | |
| --- | --- | --- | --- |
|  | **Persistent ARDS** | **RIARDS** | **Total** |
| **All Included Cohort** | **N=169** | **N=46** | **N=215** |
| IL-8, n (%) | 0 (0) | 0 (0) | 0 (0) |
| IL-6, n (%) | 2 (1) | 1 (2) | 3 (1) |
| IL-10, n (%) | 6 (4) | 2 (4) | 8 (4) |
| TNF alpha, n (%) | 6 (4) | 2 (4) | 8 (4) |
| IL-1Beta, n (%) | 6 (4) | 2 (4) | 8 (4) |
| IFN gamma, n (%) | 4 (2) | 0 (0) | 4 (2) |
| CX3CL1, n (%) | 6 (4) | 2 (4) | 8 (4) |
| sTNFR-1, n (%) | 0 (0) | 0 (0) | 0 (0) |
| sRAGE, n (%) | 0 (0) | 0 (0) | 0 (0) |
| Ang-2, n (%) | 0 (0) | 0 (0) | 0(0) |
| ICAM, n (%) | 0 (0) | 1 (2) | 1 (0.5) |
| PAI-1, n (%) | 3 (2) | 1 (2) | 4 (2) |
| Protein C, n (%) | 0 (0) | 2 (4) | 2 (1) |
| **Sensitivity Analysis (severe ARDS)** | **N=79** | **N=7** | **N=86** |
| IL-8, n (%) | 0 (0) | 0 (0) | 0 (0) |
| IL-6, n (%) | 1 (1) | 0 (0) | 1 (1) |
| IL-10, n (%) | 4 (5) | 0 (0) | 4 (5) |
| TNF alpha, n (%) | 4 (5) | 0 (0) | 4 (5) |
| IL-1Beta, n (%) | 4 (5) | 0 (0) | 4 (5) |
| IFN gamma, n (%) | 3 (4) | 0 (0) | 3 (4) |
| CX3CL1, n (%) | 4 (5) | 0 (0) | 4 (5) |
| sTNFR-1, n (%) | 0 (0) | 0 (0) | 0 (0) |
| sRAGE, n (%) | 0 (0) | 0 (0) | 0 (0) |
| Ang-2, n (%) | 0 (0) | 0 (0) | 0 (0) |
| ICAM, n (%) | 0 (0) | 0 (0) | 0 (0) |
| PAI-1, n (%) | 2 (3) | 0 (0) | 2 (3) |
| Protein C, n (%) | 0 (0) | 0 (0) | 0 (0) |
| **Sensitivity Analysis (hyperinflammatory ARDS)** | **N=82** | **N=10** | **N=92** |
| IL-8, n (%) | 1 (1) | 0 (0) | 1 (1) |
| IL-6, n (%) | 1 (1) | 1 (10) | 2 (2) |
| IL-10, n (%) | 1 (1) | 0 (0) | 1 (1) |
| TNF alpha, n (%) | 1 (1) | 0 (0) | 1 (1) |
| IL-1Beta, n (%) | 1 (1) | 0 (0) | 1 (1) |
| IFN gamma, n (%) | 1 (1) | 0 (0) | 1 (1) |
| CX3CL1, n (%) | 1 (1) | 0 (0) | 1 (1) |
| sTNFR-1, n (%) | 1 (1) | 0 (0) | 1 (1) |
| sRAGE, n (%) | 1 (1) | 0 (0) | 1 (1) |
| Ang-2, n (%) | 1 (1) | 0 (0) | 1 (1) |
| ICAM, n (%) | 0 (0) | 1 (10) | 1 (1) |
| PAI-1, n (%) | 2 (2) | 1 (10) | 3 (3) |
| Protein C, n (%) | 0 (0) | 1 (10) | 1 (1) |
| **IL-8**: Interleukin-8; **IL-6**: Interleukin-6; **TNF alpha**: tumor necrosis factor alpha; **IL-10**: Interleukin-10; **IFN gamma**: interferon gamma; **IL-1Beta**: interleukin-1 Beta; **CX3CL1**: gene encoding chemokine ligand 1; **sTNFR-1**: soluble tumor necrosis factor receptor-1; **sRAGE**: soluble receptor for advanced glycation end-products; **Ang-2**: angiopoietin-2; **ICAM-1**: intercellular adhesion molecule 1; **PAI-1**: plasminogen activator inhibitor 1. | | | |

**Table E14: Missing values, comorbidities and concomitant medical conditions.**

| **Count of Missing Values: Comorbidities and Concomitant Medical Conditions** | | | | | |
| --- | --- | --- | --- | --- | --- |
|  | **Persistent ARDS** | **RIARDS** | | **Total** | |
| **All Included Cohort** | **N=169** | **N=46** | | **N=215** | |
| Cirrhosis, n (%) | 0 (0) | 0 (0) | | 0 (0) | |
| Acquired Immunodeficiency Syndrome, n (%) | 0 (0) | 0 (0) | | 0 (0) | |
| Obstructive Sleep Apnea, n (%) | 0 (0) | 0 (0) | | 0 (0) | |
| Human Immunodeficiency Virus, n (%) | 2 (1) | 0 (0) | | 2 (1) | |
| End Stage Renal Disease, n (%) | 0 (0) | 0 (0) | | 0 (0) | |
| Other immunosuppressive state, n (%) | 0 (0) | 0 (0) | | 0 (0) | |
| Coronary Artery Disease, n (%) | 0 (0) | 0 (0) | | 0 (0) | |
| Interstitial Lung Disease, n (%) | 0 (0) | 0 (0) | | 0 (0) | |
| Chronic Kidney Disease, n (%) | 0 (0) | 0 (0) | | 0 (0) | |
| Chronic Obstructive Pulmonary Disease, n (%) | 0 (0) | 0 (0) | | 0 (0) | |
| Diastolic dysfunction, n (%) | 76 (45) | 19 (41) | | 95 (44) | |
| Hypertension, n (%) | 0 (0) | 0 (0) | | 0 (0) | |
| Multiple Myeloma, n (%) | 1 (1) | 0 (0) | | 1 (1) | |
| Leukemia, n (%) | 1 (1) | 0 (0) | | 1 (1) | |
| Alcohol Use, n (%) | 0 (0) | 0 (0) | | 0 (0) | |
| Left ventricular dysfunction, n (%) | 23 (14) | 4 (9) | | 27 (13) | |
| Restrictive Lung Disease, n (%) | 0 (0) | 0 (0) | | 0 (0) | |
| Solid Tumor, n (%) | 0 (0) | 0 (0) | | 0 (0) | |
| On immunosuppressive drugs, n (%) | 0 (0) | 0 (0) | | 0 (0) | |
| Right ventricular dysfunction, n (%) | 23 (14) | 4 (9) | | 27 (13) | |
| Cerebrovascular Disease, n (%) | 0 (0) | 0 (0) | | 0 (0) | |
| Asthma, n (%) | 0 (0) | 0 (0) | | 0 (0) | |
| Solid Organ Transplant, n (%) | 1 (1) | 0 (0) | | 1 (1) | |
| Home Oxygen, n (%) | 0 (0) | 0 (0) | | 0 (0) | |
| Outpatient Dialysis, n (%) | 2 (1) | 0 (0) | | 2 (1) | |
| Diabetes, n (%) | 1 (1) | 0 (0) | | 1 (1) | |
| Tobacco Smoking, n (%) | 0 (0) | 0 (0) | | 0 (0) | |
| Lung Transplant, n (%) | 0 (0) | 0 (0) | | 0 (0) | |
| Lymphoma, n (%) | 1 (1) | 0 (0) | | 1 (1) | |
| Lung Cancer, n (%) | 0 (0) | 0 (0) | | 0 (0) | |
| Pancreatitis, n (%) | 0 (0) | 0 (0) | | 0 (0) | |
| Thyroid Disease, n (%) | 1 (1) | 0 (0) | | 1 (1) | |
| Hypertensive Crisis, n (%) | 0 (0) | 0 (0) | | 0 (0) | |
| Acute Renal Failure, n (%) | 0 (0) | 0 (0) | | 0 (0) | |
| Acute Renal Failure Requiring Renal Replacement Therapy, n (%) | 32 (19) | 4 (9) | | 36 (17) | |
| Volume overload, n (%) | 0 (0) | 0 (0) | | 0 (0) | |
| **Sensitivity Analysis (severe ARDS)** | **N=79** | | **N=7** | | **N=86** |
| Diastolic dysfunction, n (%) | 43 (54) | | 2 (29) | | 45 (52) |
| Left ventricular dysfunction, n (%) | 15 (19) | | 0 (0) | | 15 (17) |
| Right ventricular dysfunction, n (%) | 15 (19) | | 0 (0) | | 15 (17) |
| Acute Renal Failure Requiring Renal Replacement Therapy, n (%) | 18 (23) | | 0 (0) | | 18 (21) |
| **Sensitivity Analysis (severe ARDS)** | **N=82** | | **N=10** | | **N=92** |
| Diastolic dysfunction, n (%) | 43 (52) | | 6 (60) | | 49 (53) |
| Left ventricular dysfunction, n (%) | 16 (20) | | 1 (10) | | 17 (18) |
| Right ventricular dysfunction, n (%) | 16 (20) | | 1 (10) | | 17 (18) |
| Acute Renal Failure Requiring Renal Replacement Therapy, n (%) | 19 (23) | | 0 (0) | | 19 (21) |
