# Supplementary material for: Rapidly improving ARDS differs clinically and biologically from persistent ARDS
Source: Crit Care. 2024 Apr 22;28:132. doi: 10.1186/s13054-024-04883-6 (PMC11034037; doi:10.1186/s13054-024-04883-6)
Supplement: Supplementary file 2 — Additional file 2. Supplementary Methods. This section provides additional information regarding enrollment of participants in our cohort and review of clinical charts. We also provide details on the collection and processing of plasma biomarkers. Finally, we explain how we calculated ICU-free days, ventilator-free days and hospital mortality. [file 13054_2024_4883_MOESM2_ESM.docx]

**Online Data Supplement – TEXT**

**Rapidly Improving ARDS Differs Clinically and Biologically From Persistent ARDS**

**Authors**

Patricia L Valda Toro MD MS^1^, Andrew Willmore ^2^, Nelson E Wu MPH^2^, Kevin L Delucchi PhD^4^, Alejandra Jauregui^2^, Pratik Sinha MD PhD^7^, Kathleen D Liu MD PhD^2,3^, Carolyn M Hendrickson MD MPH^2,3^, Aartik Sarma MD^2^, Lucile P A Neyton PhD^2^, Aleksandra Leligdowicz MD PhD^8^, Charles R Langelier MD PhD^5, 6^, Hanjing Zhuo MPH^2^, Chayse Jones^2^, Kirsten N Kangelaris MD^9^, Antonio D Gomez MD^2,3^, Michael A Matthay MD^2,3^, Carolyn S Calfee MD MAS^2,3^

**Corresponding autor:** Patricia L. Valda Toro MD MS
 [patricia.valdatoro@pennmedicine.upenn.edu](mailto:patricia.valdatoro@pennmedicine.upenn.edu)

**SUPPLEMENTARY METHODS**

**Participants**

Patients were enrolled in the Early Assessment of Renal and Lung Injury (EARLI) cohort from November 2008 to May 2018. EARLI (10-02852) was approved by the University of California, San Francisco Institutional Review Board (IRB). Consent was obtained from patients or their surrogates. A waiver was provided if patients were unable to consent with no identified surrogates or if the patient passed away prior to being approached for consent.

Patients were eligible for enrollment in EARLI once an ICU admission had been requested by the attending team. PaO2: FiO2 ratio was used if available for the diagnosis of ARDS; otherwise SpO2:FiO2 was used. Patients with trauma were excluded from this cohort given that one of the enrollment sites (University of California San Francisco Medical Center) is not designated a trauma center. This study analyzed data from 2008-2018; therefore, it does not include patients infected with COVID-19.

**Biomarker Assays**

Plasma samples were collected within 24 hours of enrollment. After collection, blood samples were processed and plasma stored at -80°C until batch quantification. Plasma samples frozen at baseline were thawed for protein biomarker quantification. Samples were frozen in small aliquots to minimize freeze-thaw cycles. Singleplex assays were used for the quantification of protein C (Helena Laboratories; Beaumont, TX) and plasminogen activator inhibitor-1 (PAI-1, R&D systems; Minneapolis, MN) and were measured as duplicates. All other biomarkers, including interleukin-8 (IL-8), interleukin-6 (IL6), and intercellular adhesion molecule-1 (ICAM-1), were measured using a multiplex ELISA (R&D systems; Minneapolis, MN). For all biomarkers, a thorough quality control procedure was followed for each analyzed plate. Measures of plasma biomarkers were available for the majority of patients as outlined in Table E9.

**Comorbidities and Concomitant Medical Conditions Review**

Patients’ comorbidities were obtained from manual review of clinical charts. Review was performed independently by study coordinators and trained physicians in three different occasions. Reviewers were blinded to categorization of patients as rapidly improving ARDS versus persistent ARDS. Data from each independent review was later compared. Discrepancies in data extracted prompted a fourth review of specific comorbidities. Twenty-eight patients admitted before the year 2010 had limited charts available in our current electronic medical records at time of fourth chart review; absent data for these patients is outlined in Table E10 in the online supplement material.

Coronary artery disease was obtained by review of clinical charts and review of left heart catheterization reports, if available. Systolic or diastolic ventricular dysfunction was similarly obtained by review of clinical charts and review of echocardiogram reports that were closest to the date of ICU admission. Echocardiograms reports were missing for several patients mostly admitted prior to the year 2010 as outlined in Table E10. Tachycardia during the echocardiogram precluded the assessment of diastolic dysfunction in several patients; for this reason, presence of diastolic dysfunction is missing for several patients as outlined in Table E10.

Evaluation of immunosuppression was divided as either immunosuppression secondary to medications (including chemotherapies, immunomodulators and steroids) or the presence of other immunocompromised state. The latter included immunosuppression from medications as well as immunosuppression from HIV positivity, AIDS, diabetes, cirrhosis, malignancy (solid or liquid) or immunodeficiencies (i.e. common variable immunodeficiency).

Concomitant medical conditions that may predispose to cardiogenic pulmonary edema were also analyzed via review of clinical notes. More specifically, presence of (i) hypertensive crisis, (ii) volume overload or (iii) acute renal failure requiring renal replacement therapy, was queried by searching the likelihood of this diagnosis based on the clinical judgment of the treating physician as reported in medical notes.

**ICU-free days, ventilator-free days, and hospital mortality**

ICU-free days were calculated as the difference between the ICU discharge date and the ICU admit date, subtracted from 28 days. Patients who were either discharged from the ICU after day 28 or expired during the 28-day period, were assigned zero ICU-free days.

Ventilator-free days (VFD) were calculated at 28 days. Zero VFD were assigned to patients who remained intubated after day 28 or died between day 0 and day 28.

Hospital mortality was calculated at 28 days. Two patients known to die within 28 days of admission but after hospital discharge were noted. All other patients who died within 28 days of admission died within the same hospitalization.
