# Supplementary figures and images for: Rapidly improving ARDS differs clinically and biologically from persistent ARDS
Source: Crit Care. 2024 Apr 22;28:132. doi: 10.1186/s13054-024-04883-6 (PMC11034037; doi:10.1186/s13054-024-04883-6)

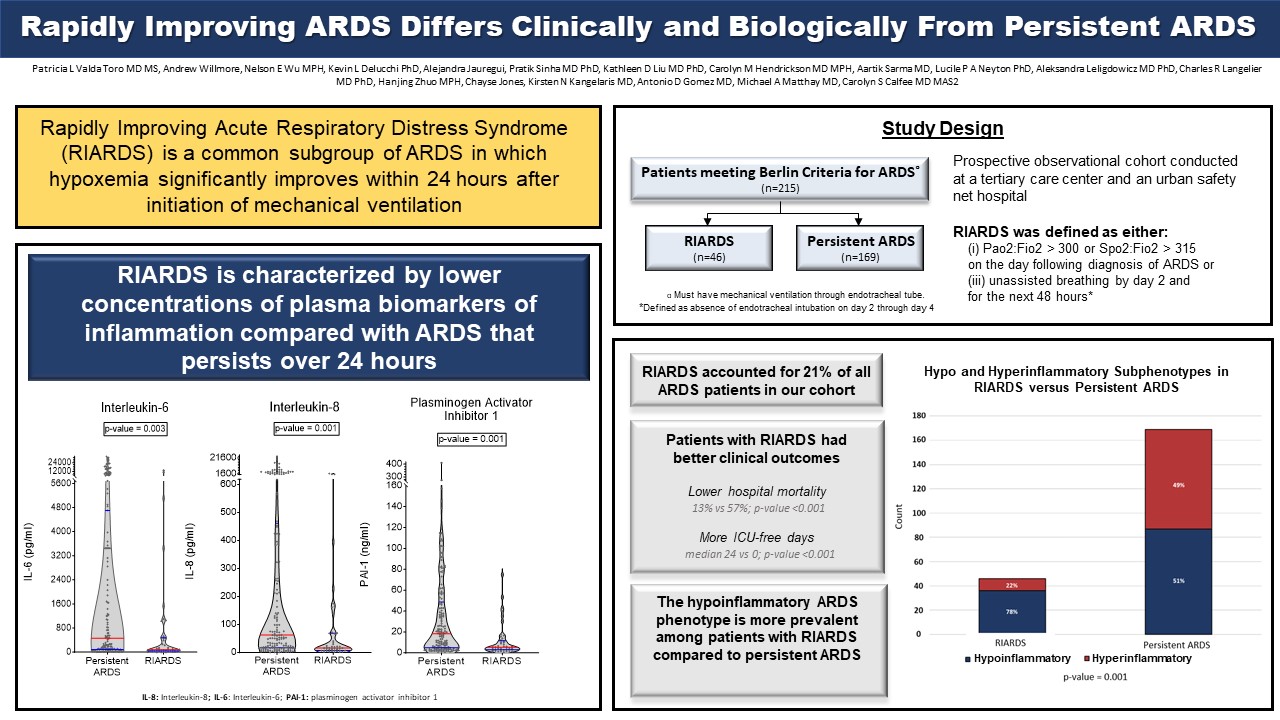

Supplement: Supplementary file 3 — Additional file 3. Visual Abstract. Rapidly improving acute respiratory distress syndrome (RIARDS) is a subgroup of ARDS in which hypoxemia significantly improves within 24 hours after initiation of mechanical ventilation. We analyzed data in patients with RIARDS (defined as (i) PaO2:FiO2>300 or (ii) SpO2:FiO2>315 on the day following diagnosis of ARDS or (iii) unassisted breathing by day 2 and for the next 48 hours) and in patients with persistent ARDS. Patients with RIARDS had better clinical outcomes compared to those with persistent ARDS, with lower hospital mortality and more ICU-free days. Plasma levels of inflammatory markers were significantly lower among patients with RIARDS. The hypoinflammatory phenotype of ARDS was more common among patients with RIARDS. [file 13054_2024_4883_MOESM3_ESM.jpg]
